# Structure of the *Neisseria* Adhesin Complex Protein (ACP) and its role as a novel lysozyme inhibitor

**DOI:** 10.1371/journal.ppat.1006448

**Published:** 2017-06-29

**Authors:** María Victoria Humbert, Amaka Marian Awanye, Lu-Yun Lian, Jeremy P. Derrick, Myron Christodoulides

**Affiliations:** 1 Neisseria Research, Molecular Microbiology, Academic Unit of Clinical and Experimental Sciences, Sir Henry Wellcome Laboratories, University of Southampton Faculty of Medicine, Southampton, United Kingdom; 2 Faculty of Biology, Medicine and Health, University of Manchester, Michael Smith Building, Oxford Road, Manchester, United Kingdom; 3 NMR Centre for Structural Biology, Institute of Integrative Biology, University of Liverpool, Liverpool, United Kingdom; University of Oxford, UNITED KINGDOM

## Abstract

Pathogenic and commensal *Neisseria* species produce an Adhesin Complex Protein, which was first characterised in *Neisseria meningitidis* (Nm) as a novel surface-exposed adhesin with vaccine potential. In the current study, the crystal structure of a recombinant (r)Nm-ACP Type I protein was determined to 1.4 Å resolution: the fold resembles an eight-stranded β-barrel, stabilized by a disulphide bond between the first (Cys38) and last (Cys121) β-strands. There are few main-chain hydrogen bonds linking β4-β5 and β8-β1, so the structure divides into two four-stranded anti-parallel β-sheets (β1-β4 and β5-β8). The computed surface electrostatic charge distribution showed that the β1-β4 sheet face is predominantly basic, whereas the β5-β8 sheet is apolar, apart from the loop between β4 and β5. Concentrations of rNm-ACP and r*Neisseria gonorrhoeae*-ACP proteins ≥0.25 μg/ml significantly inhibited by ~80–100% (P<0.05) the *in vitro* activity of human lysozyme (HL) over 24 h. Specificity was demonstrated by the ability of murine anti-*Neisseria* ACP sera to block ACP inhibition and restore HL activity. ACP expression conferred tolerance to HL activity, as demonstrated by significant 3–9 fold reductions (P<0.05) in the growth of meningococcal and gonococcal *acp* gene knock-out mutants in the presence of lysozyme. In addition, wild-type *Neisseria lactamica* treated with purified ACP-specific rabbit IgG antibodies showed similar fold reductions in bacterial growth, compared with untreated bacteria (P<0.05). Nm-ACPI is structurally similar to the MliC/PliC protein family of lysozyme inhibitors. However, *Neisseria* ACP proteins show <20% primary sequence similarity with these inhibitors and do not share any conserved MliC/PliC sequence motifs associated with lysozyme recognition. These observations suggest that *Neisseria* ACP adopts a different mode of lysozyme inhibition and that the ability of ACP to inhibit lysozyme activity could be important for host colonization by both pathogenic and commensal *Neisseria* organisms. Thus, ACP represents a dual target for developing *Neisseria* vaccines and drugs to inhibit host-pathogen interactions.

## Author summary

The genus *Neisseria* contains two major human pathogens: *N*. *meningitidis* (Nm) causes meningitis and sepsis, and *N*. *gonorrhoeae* (Ng) causes the sexually transmitted disease gonorrhoea. In addition, the genus contains a larger number of commensal organisms, including *N*. *lactamica* (Nl). Common to all of these organisms is the ability to colonize exposed mucosal epithelia. Recently, we identified a novel surface-exposed adhesin in *Neisseria* spp., the Adhesin Complex Protein (ACP), which was capable also of generating a functional bactericidal antibody response in mice. In the current study, we have determined the crystal structure of a recombinant (r)Nm-ACP and shown that it shares structural homology to bacterial lysozyme inhibitors. We demonstrate that *Neisseria* ACP functions as an inhibitor of mammalian lysozyme but the mechanism appears to be different from other bacterial family lysozyme inhibitors. Expression of ACP enables *Neisseria* spp. to tolerate human lysozyme. We propose that ACP-mediated inhibition of lysozyme activity could be important for host colonization by both pathogenic and commensal *Neisseria* organisms and that ACP represents not only a target for developing *Neisseria* vaccines but also drugs to inhibit host-pathogen interactions.

## Introduction

Lysozymes are ubiquitous enzymes with N-acetylmuramoyl hydrolase activities, which hydrolyse the bacterial cell wall polymer peptidoglycan (PG). PG is the major structural component of the bacterial cell wall: its major function is to provide resistance against turgor pressure and its cleavage results in bacteriolysis [1]. Therefore, host lysozymes are an important component of innate immunity, contributing to a first line of defence against bacterial colonization or infection. In humans, C-type lysozyme can be found on all mucosal surfaces and secretions [2, 3], including the respiratory airway [4], the digestive tract [5], milk [6] as well as in serum [7].

Bacteria engaging in commensal or pathogenic interactions with a human or animal host have evolved various strategies to evade lysozymal activity. One mechanism of lysozymal resistance that both Gram-positive and Gram-negative bacteria use is PG modification, which has been demonstrated by several pathogens, including *Streptococcus pneumoniae*, *Staphylococcus aureus*, *Listeria monocytogenes*, *Neisseria meningitidis* and *Neisseria gonorrhoeae* [8]. A second mechanism to protect bacteria against host lysozyme involves the production of lysozyme inhibitors. Expression of lysozyme inhibitors likely contributes to bacterial colonization and infection. These enzymes can also function as important mediators in bacteria–bacteria interactions, by modulating the generation of PG fragments and by providing protection against other bacterial lysozymes in bacterial competition [1]. Furthermore, bacterial lysozyme inhibitors have been shown to control autolysis by inhibiting the lytic activity of transglycoslyases, which are enzymes involved in the biosynthesis and maintenance of PG [9]. To date, these lysozyme inhibitors have been identified only in Gram-negative bacteria such as *Escherichia coli* [10, 11], *Salmonella enteriditis* [11], *Pseudomonas aeruginosa* [11, 12] and, more recently, *Mycobacterium tuberculosis* [13], among others [1]. These inhibitors are located either in the periplasm or anchored to the luminal face of the outer membrane (OM). However, lysozyme inhibitor(s) for several important Gram-negative pathogens, such as *Legionella* spp., *Campylobacter* spp., or *Neisseria* spp. have not been reported to date. For these pathogens, PG modification has been described [14, 15], although the existence of other mechanisms to counter host lysozymal activity cannot be excluded.

Members of the genus *Neisseria* colonize mucosal surfaces: *Neisseria meningitidis* (Nm, meningococcus), the causative organism of meningococcal meningitis and sepsis [16], and the commensal organism *Neisseria lactamica*, both colonize the human nasopharyngeal mucosal epithelium [17]. *Neisseria gonorrhoeae* (Ng, gonococcus) is closely related to the meningococcus, but colonizes the mucosal epithelium of the reproductive tract of both men and women and causes the sexually transmitted disease gonorrhoea [18, 19]. Recently, we described a novel adhesin termed the Adhesin Complex Protein (ACP—NMB2095/NEIS2075), a conserved protein found in the OM of meningococci, gonococci, *N*. *lactamica* and all other *Neisseria* spp. [20]. We identified that the majority of sequenced meningococcal isolates contained one of two distinct Nm-ACP type proteins: Nm-ACPI and Nm-ACPII, encoded by Alleles 1 and 2 respectively. Nm-ACP acts as a minor adhesin, mediating the adherence of meningococci principally with epithelial cells. A recombinant Nm-ACPI protein was also capable of inducing cross-protective bactericidal antibodies, suggesting that it might be a potential vaccine candidate for inclusion in second-generation meningococcal vaccines. In the current study, we have examined further the biological properties of *Neisseria* ACP proteins by determination of the crystal structure of Nm-ACPI. We show that it has some structural similarity to the MliC/PliC protein family, which are membrane-bound or periplasmic inhibitors of C-type lysozyme. We also present data that show that *Neisseria* ACP acts as a lysozyme inhibitor, fulfilling this ubiquitous function for which no protein has been reported so far for any of the *Neisseria* species, although the mechanism of inhibition appears to be different from the MliC/PliC family.

## Results

### Crystal structure of *Neisseria menigitidis*-Adhesin Complex Protein Type I (Nm-ACPI)

We previously described expression of the Nm-ACPI protein from *N*. *meningitidis* strain MC58 in *E*.*coli* as a recombinant (r) full length protein (124 residues) with an N-terminal hexa-histidine tag linked to the leader peptide. The molecular mass (*Mr*) of this expressed protein was ~17.8 kDa; the protein was insoluble and was therefore purified under denaturing conditions [20]. Here, we optimized the expression of rNm-ACPI protein by inserting the full length sequence for expression and export to the periplasm; the signal peptide sequence is cleaved by the *E*. *coli* signal peptidase and the periplasm provides an oxidizing environment for the formation of a disulphide bond (see below). rNm-ACPI was purified in soluble form by serial column chromatography steps, to give a homogeneous product that eluted as a single peak from a size exclusion chromatographic column, at an elution volume consistent with a monomer (S1A and S1B Fig). Mass spectrometry was used to verify that the predicted signal peptide was cleaved after export to the periplasm and gave a mass of 12,306 Da, consistent with a mature polypeptide of 103 residues plus the C-terminal hexa-histidine tag.

**Fig 1:**
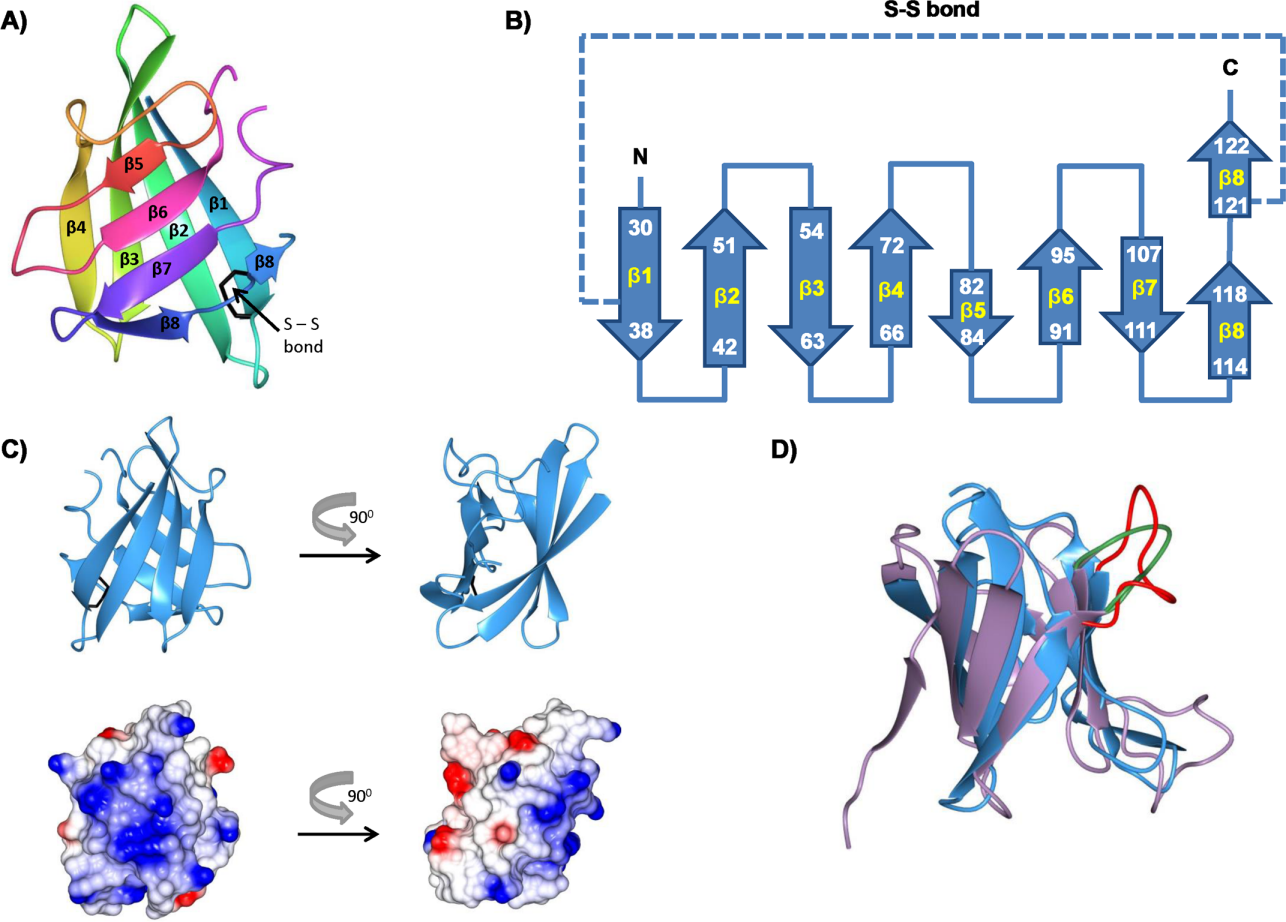
Structure of rNm-ACPI and comparison with the MliC/PliC canonical fold. **A)** Ribbon structure, showing the eight β-strands and disulphide bond. **B)** Topology diagram showing residue ranges in each β-strand, and the disulfide bond between Cys38 and Cys121 residues. **C)** Two orthogonal views of rNm-ACPI with the corresponding electrostatic surface in the same orientations below. **D)** Structural superposition of rNm-ACPI (blue) with PliC from *Brucella abortus* (Ba-PliC; PDB 4ML7; lilac), as a representative of the MliC/PliC lysozyme inhibitor family. Loop 4 is highlighted in red for rNm-ACPI and green for Ba-PliC.Images were constructed using CCP4MG [58].

Crystallization trials were initiated on the purified protein, and native data were collected from a single crystal to 1.4 Å resolution (Table 1 and Table 2). The structure was solved by single wavelength anomalous diffraction from iodide ions, following a soak of a crystal in 0.4 M potassium iodide. The overall fold of Nm-ACPI is close to an 8-stranded β-barrel, stabilized by a disulphide bond between cysteine residues on the first and last β-sheet (Fig 1A and 1B). However, there are few main-chain hydrogen bonds linking β4-β5 and β8-β1, meaning that the structure divides into two four-stranded anti-parallel β-sheets (β1-β4 and β5-β8; Fig 1C). The interaction between β8 and β1 is stabilised by the disulphide bridge between Cys38 and Cys121. The computed surface electrostatic charge distribution showed that the face of the β1-β4 sheet is predominantly basic, whereas the β5-β8 sheet is more apolar, apart from the loop between β4 and β5 (Fig 1C).

**Table 1:** Crystal structure of rNm-ACPI: Data Collection.

**Data collection**	**Native**	**Iodine**	
Space group	*P3*_*2*_*21*	*P3*_*2*_*21*	
Unit cell parameters	a = b = 58.5Å, c = 51.6Å		a = b = 56.4Å, c = 51.1Å
X-ray source and wavelength (Å)	DLS^a^ IO4-1 (0.9282)		DLS IO3 (1.50)
Number of crystals	1		1
Resolution range (Å)	50.64–1.40 (1.42–1.40)^b^		51.12–1.64 (1.68–1.64)
Multiplicity	5.9 (6.0)		68.7 (9.2)
Significance (<I>/σI)	13.5 (1.8)		52.9 (1.6)
Total reflections	120,245 (5,992)		734,657 (2,706)
No. unique reflections	20,461 (1,001)		10,689 (295)
Completeness (%)	99.9 (100)		90 (35.3)
*R*_*merge*_ (%)^c^	5.4 (99.7)		6.0 (120)
*R*_*p*.*i*.*m*._ (%)^d^	3.6 (66.8)		0.7 (42.9)
Anomalous completeness (%)			87.7 (21.4)
Anomalous multiplicity			35.2 (5.3)
Anomalous slope			1.505

^a^Diamond Light Source

^b^values in parentheses refer to the outer resolution shell

^c^ $R_{merge}=\frac{\sum_{hkl}\sum_{j}\left|I_{hkl,j}-\langle I_{hkl}\rangle \right|}{\sum_{hkl}\sum_{j}I_{hkl,j}}$

^d^ $R_{p.i.m.}=\frac{\sum_{hkl}\sqrt{\frac{1}{n-1}}\sum_{j=1}^{n}\left|I_{hkl,j}-\langle I_{hkl}\rangle \right|}{\sum_{hkl}\sum_{j}I_{hkl,j}}$

**Table 2:** Crystal structure of rNm-ACPI: Refinement Statistics.

**Refinement Statistics**	
R_cryst_	16.4
R_free_ (5.0% data)	20.2
Non-hydrogen atoms	
All	754
Water	31
Mean overall B (Å^2^)	28.6
RMSD from ideal values	
Bond distance (Å)	0.021
Bond angle (degrees)	2.0

### Nm-ACPI is structurally homologous to MliC but does not share any conserved MliC/PliC motifs

**Fig 2:**
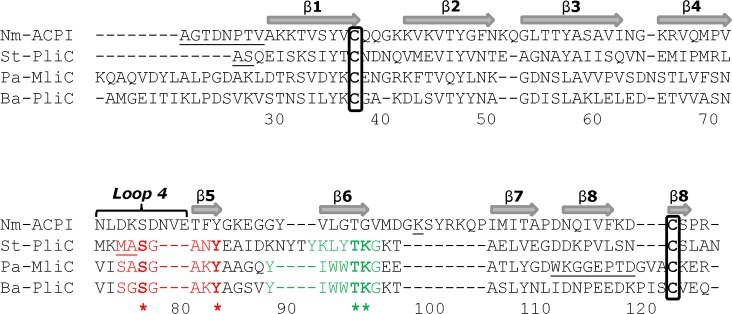
Structure-based sequence alignment of Nm-ACPI with the MliC/PliC family of lysozyme inhibitor proteins. The sequence of ACP from *Neisseria meningitidis* serogroup B strain MC58 (E6MZT7) was aligned with those for St-PliC from *Salmonella typhimurium* (Q8ZPY8); Pa-MliC from *Pseudomonas aeruginosa* (Q91574) and Ba-PliC from *Brucella abortus* (Q57ES7) based on structural superpositions carried out using CCP4MG [58]. β-strands β1 – β8 are indicated by the shaded arrows, and are based on Nm-ACPI structure. Conserved cysteine residues of the mature proteins are blocked in black. Numbering follows the Nm-ACPI sequence. Underlined residues had weak or no density and were not included in the crystal structures. Sequence motifs involved in the interaction of *P*. *aeruginosa* MliC with Hewl are in red and green, and conserved residues within these regions are marked in bold and identified by similarly coloured asterisks (*).

Using the DALI fold comparison server [21], we searched for proteins with similar structures: top ‘hits’ from the search were PliC from *Salmonella typhimurium* (St-PliC), MliC from *Pseudomonas aeruginosa* (Pa-MliC) and PliC from *Brucella abortus* (Ba-PliC). These form part of the MliC/PliC family of lysozyme inhibitor proteins from Gram-negative bacteria [22–24]. All three structures superposed onto Nm-ACPI with r.m.s. deviations of less than 2.0Å, and all preserved the same eight stranded β-fold (Fig 1D), although sequence identity with Nm-ACPI was less than 20% in each case. A structure-based sequence alignment showed that the disulphide bridge between β1 and β8 was preserved in each case, although the sizes of the loops varied (Fig 2). In addition, we noticed that loop 4 from the MliC/PliC family proteins, which occupies the lysozyme active site, corresponded to loop 4 of Nm-ACPI, but was longer in the latter by 3 residues. The sequence motifs SxSGAxY and YxxxTKG, which were conserved in the MliC/PliC family proteins, were not retained in Nm-ACPI (Fig 2). These observations suggested potentially a different mode of Nm-ACPI binding to lysozyme.

### *Neisseria* ACP proteins function as lysozyme inhibitors

**Fig 3:**
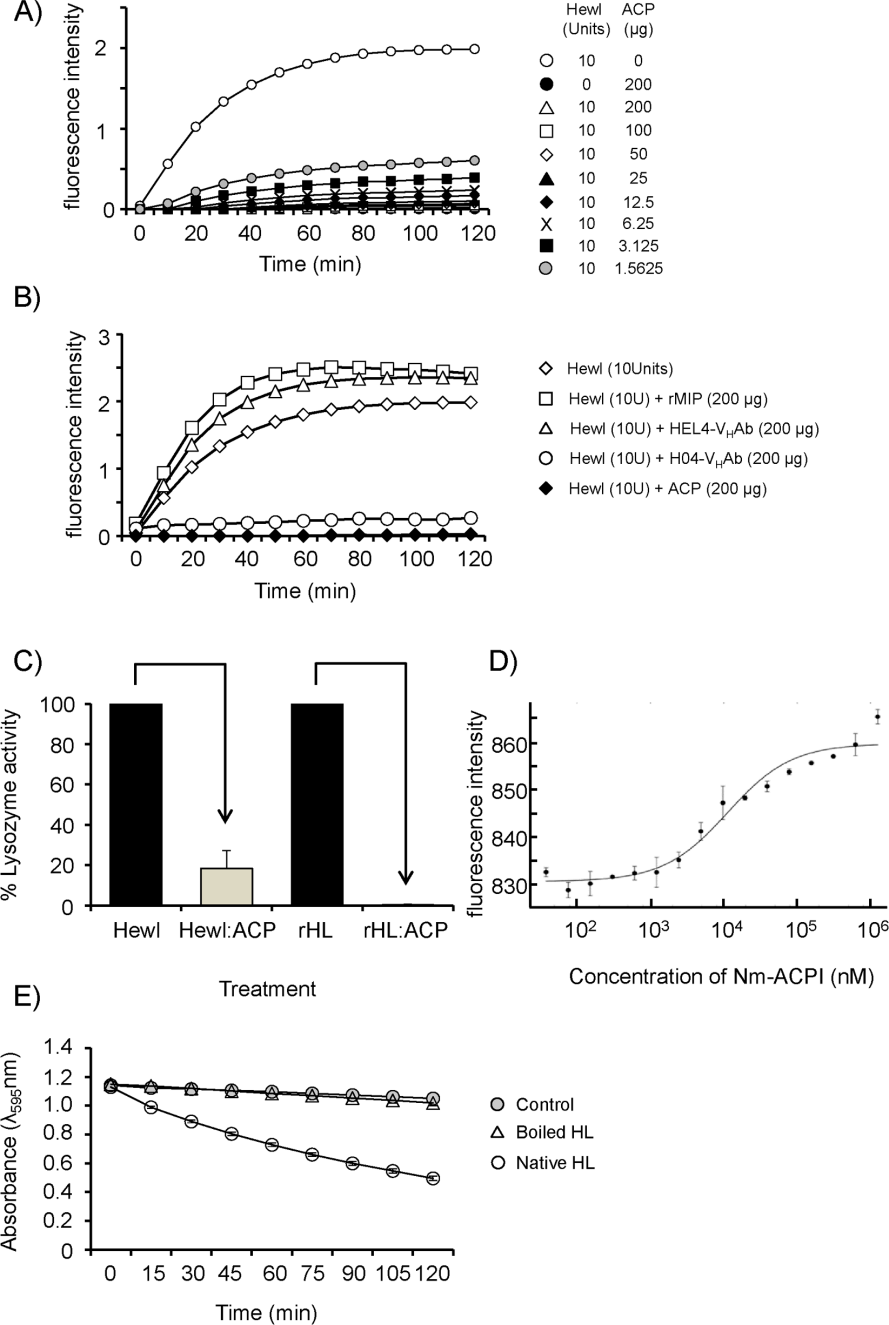
Inhibition of hen egg white lysozyme (Hewl) by rNm-ACPI. The ability of purified rNm-ACPI to suppress the hydrolytic activity of Hewl was measured using a fluorescently tagged peptidoglycan substrate from *Microccocus lysodeikticus*. **A)** Inhibition of Hewl activity by increasing concentrations of rNm-ACPI. **B)** Inhibition of Hewl activity by positive control antibody H04 VH-Ab; negative controls of antibody HEL4 VH-Ab or a heterologous purified rNm-MIP protein showed no inhibition of activity. For **A)** and **B)** the symbols represent the mean and the error bars the standard deviations of n = 2–3 independent measurements at each time point. Data were compared with a paired t-Test. **C)** Comparison of the inhibition of HL and Hewl activity by incubation with molar equivalent concentration of rNm-ACPI. The bars represent the mean and the error bars the standard deviations of n = 3 independent measurements of lysozyme activity. Data were compared with a two-sample t-Test and significant reductions denoted by the arrows (P<0.05). **D)** Binding isotherm of rNm-ACPI bound to HL analysed by MicroScale Thermophoresis (MST). rNm-ACPI was added to a solution of recombinant HL (8.3 nM) labelled with NT-647 MST dye and normalised fluorescence (F_norm_) was recorded. The fitted curve shows a *K*_*d*_ of 11 μM. **E)** Survival of *M*. *lysodiekticus* (1 mg/ml) in the presence of boiled HL. Bacterial suspensions were treated with native HL (2 μg/ml), boiled HL (2 μg/ml) or left untreated (control), and absorbance measured at intervals over 2 h. The symbols represent the mean absorbance values and the error bars represents the standard error of the mean of n = 4 independent experiments. Data were compared with a paired t-Test.

Since Nm-ACPI is similar in structure to the MliC/PliC family proteins, we used *in vitro* lysozyme inhibition assays to test the hypothesis that it could act as an inhibitor of C-type lysozyme isolated from hen egg-white (Hewl) and from human neutrophils (HL). The Hewl assay uses a *Micrococcus lysodiekticus* suspension in which the bacterial cell walls are labelled with fluorescein to such a degree that the fluorescence is quenched. The suspension acts as the lysozyme substrate, such that release of the quenched fluorophore upon hydrolysis of peptidoglycan by Hewl action gives higher fluorescence signal intensities. Addition of doses of rNm-ACPI as low as 12.5 μg/100μL assay volume, containing 10U of Hewl, led to almost complete inhibition of Hewl activity, as judged by this lysozyme assay (Fig 3A). Even at the lowest dose of rNm-ACPI tested, ~1.6μg /100μL assay volume, an ~70% reduction of Hewl activity was observed (P<0.05) (Fig 3A). As expected, treatment with a positive control antibody H04 V_H_-Ab, which binds and occupies the active site of lysozyme, inhibited Hewl activity completely, similar to rNm-ACPI treatment (P<0.05) (Fig 3B). By contrast, treatment with a negative control HEL4 V_H_-Ab, which binds to lysozyme but not at the active site, showed no inhibitory effect on Hewl activity (P>0.05) (Fig 3B). Next, the same assay was used under identical conditions to compare the inhibition of HL and Hewl activity by rNm-ACPI, using equimolar quantities of protein and lysozyme (Fig 3C): in these experiments, Hewl activity was reduced by ~82%, whereas HL activity was virtually completely eliminated (>99% reduction), reflecting a statistically significant higher efficiency of rNm-ACPI inhibition of the human enzyme (P<0.05). In addition to enzyme inhibition, the binding of rNm-ACPI to HL was analysed by MicroScale Thermophoresis (MST), a method which uses a microscopic temperature gradient combined with detection of HL, which had been pre-labelled with NT-647-NHS fluorescent dye. The data were consistent with a single binding isotherm and a *K*_*d*_ value of 11 μM (= 13.5μg/100μL assay volume) was recorded (Fig 3D). Ostensibly this relatively weak binding affinity is at odds with the more potent inhibition seen at lower concentrations in Fig 3A. However, inhibition in the enzyme assay in Fig 3A is carried out in the presence of substrate, which is not the case for the experiment in Fig 3D. It is possible that the *K*_*d*_ value of ACP for lysozyme is influenced by the presence of lysozyme substrate (*e*.*g*. if ACP also binds to cell wall components).

To exclude the possibility that lysozyme antimicrobial activity was responsible for the observed killing of *M*. *lysodiekticus*, we treated bacterial suspensions with HL that had been boiled for 1 h, a treatment that has been reported to destroy peptidoglycan hydrolase activity, whilst maintaining antimicrobial activity [25–27]. We confirmed that boiled HL had no activity against *M*. *lysodiekticus* (Fig 3E), indicating that lysozyme does not induce *M*. *lysodiekticus* cell death by antimicrobial activity of the molecule.

**Fig 4:**
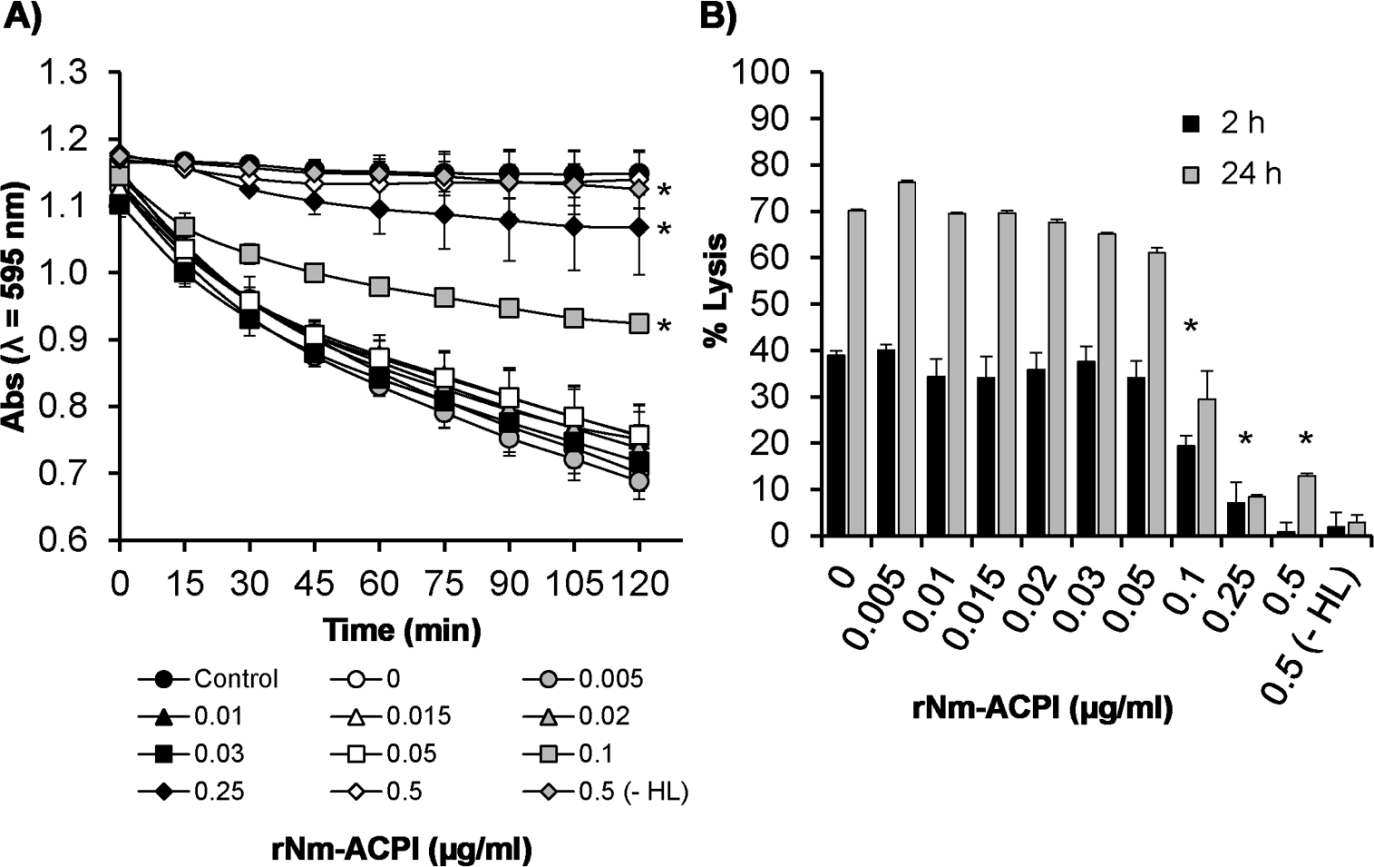
Inhibition of human neutrophil lysozyme (HL) activity of rNm-ACPI. **A)** Lysis of a 1 mg/ml *Micrococcus lysodeikticus* cell suspension (expressed as a reduction in ODλ_595_nm against time) in the absence or in the presence of increasing concentrations of rNm-ACPI and 2 μg/ml of HL. The curves represent the mean absorbance (ODλ_595_nm) and the error bars represent the corresponding standard error of the mean (SEM) of three independent experiments. Data were compared with a paired t-Test and the asterisks (*) denote significant difference (P<0.05) in ODλ_595_nm in comparison to the control treatment with HL only without rNm-ACPI (0 μg/ml). **B)** Percentage lysis of *M*. *lysodeikticus* cell suspension estimated for each condition after 2 h and 24 h incubation. *M*. *lysodeikticus* cells alone or in the presence of 0.5 μg/ml of rNm-ACPI without HL were used as negative control conditions for lysis. The positive control sample consisted of *M*. *lysodeikticus* cells in the presence of HL only. The columns represent the mean (for the same n = 3 independent experiments) and the error bars represent the corresponding SEM. Data were compared with a two-sample t-Test and the asterisks (*) denote significant difference (P<0.05) compared to the control without rNm-ACPI.

We examined also the kinetics of rNm-ACPI inhibition of HL activity by measuring lytic absorbance over time. In preliminary experiments to quantify HL concentrations that produced significant lysis, suspensions of *M*. *lysodeikticus* cells (initial OD λ_595_nm of 1–1.2, equivalent to 1 mg/ml) were treated with increasing concentrations of HL (0–5 μg/ml) and absorbance was measured every 5 min for a period of 2 h. A final HL concentration of 2 μg/ml was chosen as optimal for inducing a significant (P<0.05) linear decrease in absorbance values of >50% after 2 h incubation with 1 mg/ml *M*. *lysodeikticus* cells (S2 Fig). These assay parameters were used to examine the dose-dependent kinetics of rNm-ACPI inhibition of HL activity. A dose of 0.1 μg/ml rNm-ACPI conferred ~30–60% protection of *M*. *lysodeikticus* cells against HL lysis by 2 h (Fig 4A). Higher doses were even more effective, with ≥0.25 μg/ml of rNm-ACPI providing ~80–100% protection by 2 h. Notably, similar levels of inhibition of HL activity by rNm-ACPI were observed after 24 h incubation, with the *M*. *lysodeikticus* cells still intact after prolonged exposure to enzyme (Fig 4B). Doses of rNm-ACPI <0.1 μg/ml were not protective against HL lysis (Fig 4A and 4B) (P>0.05).

**Fig 5:**
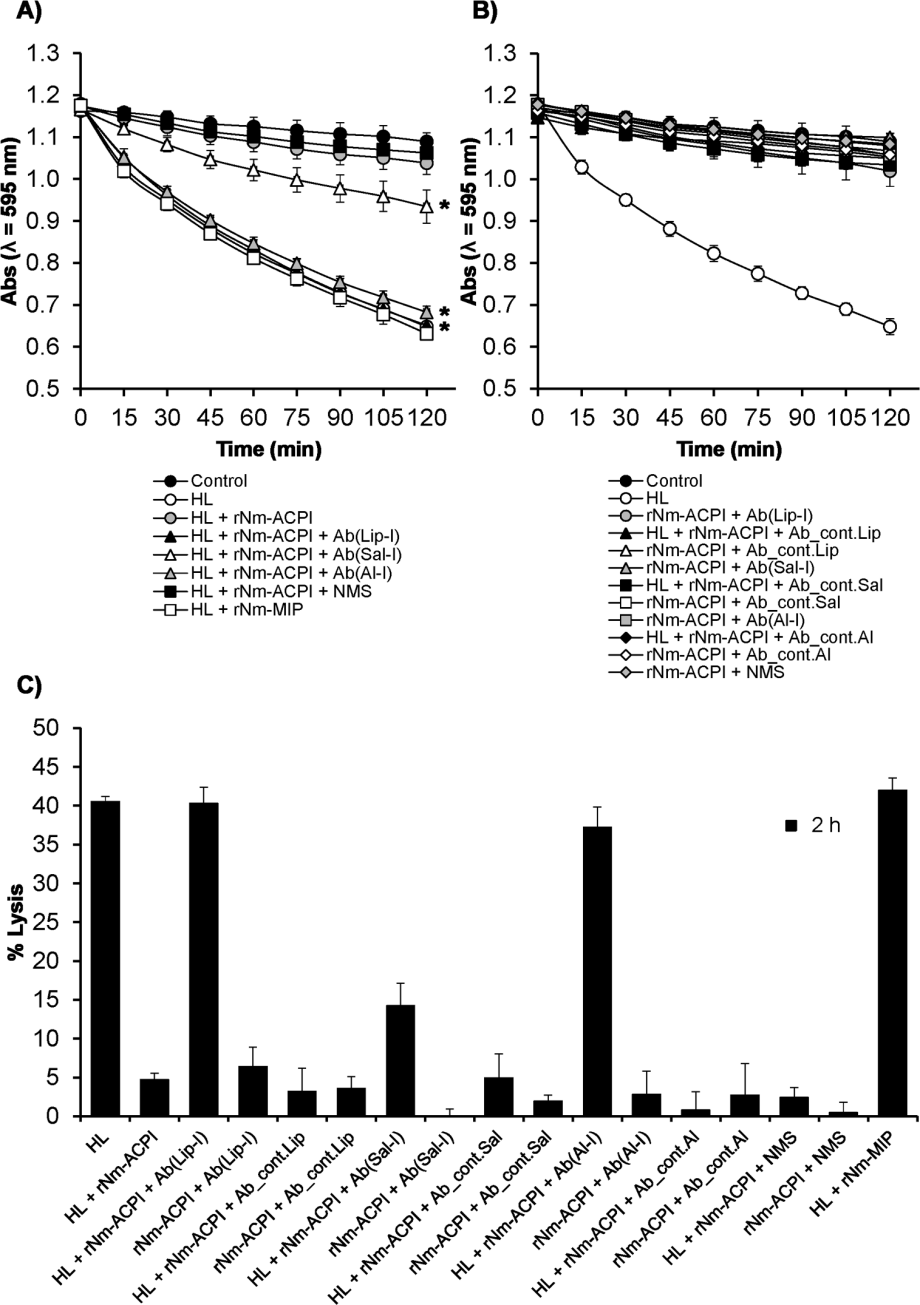
Antibodies to rNm-ACPI prevent rNm-ACPI from inhibiting HL lytic activity on *M*. *lysodeikticus* cells. HL inhibitory activity by rNm-ACPI (0.5 μg/ml) was analysed as a reduction in ODλ_595_nm (Absorbance, Abs) against time of a *M*. *lysodeikticus* cell suspension (1 mg/ml) in the presence or absence of **A)** decomplemented murine antisera raised against rNm-ACPI protein in different formulations and **B)** sera from sham immunised mice. Normal mouse serum (NMS) and addition of a recombinant heterologous rNm-MIP protein were included as negative control. The symbols represent the mean absorbance (ODλ_595_nm) (from n = 3 independent experiments) and the error bars represent the corresponding standard error of the mean (SEM). Data were compared with a paired t-Test and the asterisks (*) denote significant inhibition (P<0.05) of rNm-ACPI function by anti-rNm-ACPI sera, compared to treatment without antisera. **C)** Determination of percentage of *M*. *lysodeikticus* cell lysis for each test condition shown in **A)** and **B)** for 2 h incubation time point only. The columns represent the mean % lysis (from n = 3 independent experiments) and the error bars represent the corresponding SEM. Ab(Lip-I), Ab(Al-I) and Ab(Sal-I) refer to decomplemented, pooled (n = 5) murine sera raised against rNm-ACPI delivered in liposomes, Al(OH)_3_ or saline solution, respectively. Ab_cont.Lip, Ab_cont.Al and Ab_cont.Sal refer to the corresponding sham immunised decomplemented, pooled (n = 5) murine sera.

Next, we examined the specificity of the rNm-ACPI inhibitory activity towards HL by using murine antisera raised against native protein to restore the activity of HL. Decomplemented murine antisera raised against the protein using a variety of adjuvants as described elsewhere [20], were added to a mixture of rNm-ACPI (0.5 μg/ml), *M*. *lysodeikticus* cell suspension (1 mg/ml) and HL (2 μg/ml). Antisera generated by rNm-ACPI immunisation delivered in Al(OH)_3_ or liposome formulations significantly prevented rNm-ACPI protein from inhibiting HL activity by ≥ 95% (P<0.05) after 2 h (Fig 5A). In comparison, antisera raised to rNm-ACPI in saline prevented rNm-ACPI protein from inhibiting HL activity by ≥ 50% (P<0.05) after 2 h (Fig 5A), which is likely due to lower levels of antibody production through immunization (S3 Fig). Furthermore, sham sera or normal mouse serum (NMS) did not prevent rNm-ACPI from inhibiting HL activity (Fig 5B). The specificity of rNm-ACPI as an inhibitor of HL activity was also supported by the observation that a heterologous recombinant outer membrane protein, Nm-Macrophage Infectivity Potentiator (rNm-MIP, NMB1567/NEIS1487) did not inhibit lysis of *M*. *lysodeikticus* induced by either Hewl (Fig 3B) or HL (Fig 5A).

**Fig 6:**
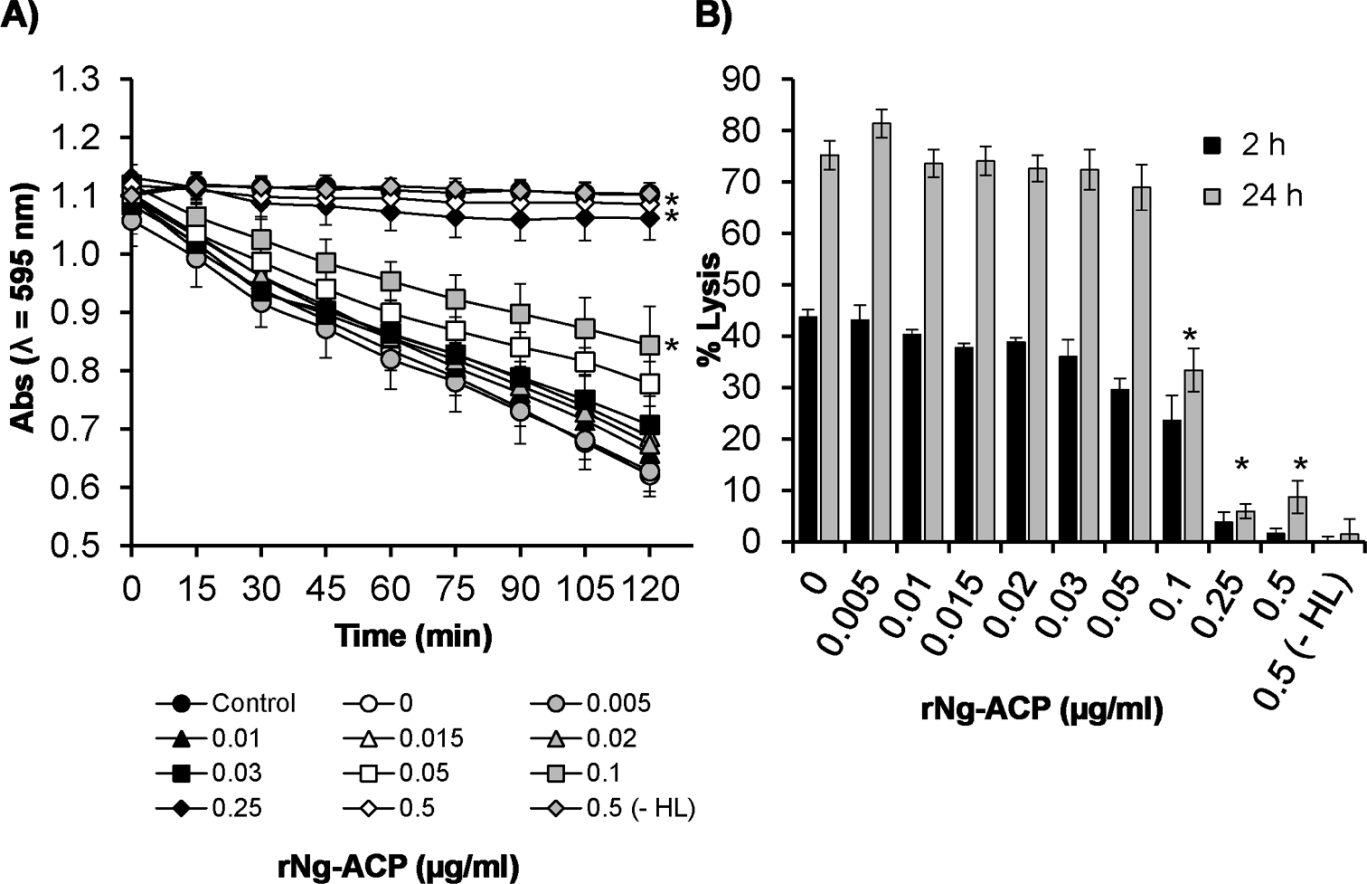
Human neutrophil lysozyme inhibitory activity of rNg-ACP. **A)** Lysis of a 1 mg/ml *Micrococcus lysodeikticus* cell suspension (expressed as a reduction in ODλ_595_nm against time) in the absence or in the presence of increasing concentrations of rNg-ACP and 2 μg/ml of HL. The curves represent the mean absorbance (ODλ_595_nm) and the error bars represent the corresponding standard error of the mean (SEM) of three independent experiments. Data were compared with a paired t-Test and the asterisks (*) denote significant difference (P<0.05) in ODλ_595_nm in comparison to the control treatment with HL only without rNg-ACP (0 μg/ml). **B)** Percentage lysis of *M*. *lysodeikticus* cell suspension estimated for each condition after 2 h and 24 h incubation. *M*. *lysodeikticus* cells alone or in the presence of 0.5 μg/ml of rNg-ACP without HL were used as negative control conditions for lysis. The positive control sample of lysis consisted of *M*. *lysodeikticus* cells in the presence of HL only. The columns represent the mean (from n = 3 independent experiments) and the error bars represent the corresponding SEM. Data were compared with a two-sample t-Test and the asterisks (*) denote significant difference (P<0.05) compared to the control without rNg-ACP.

Amino acid sequence alignment of the Nm-ACPI (encoded by Allele 1), Nm-ACPII (encoded by Allele 2) and *Neisseria gonorrhoeae* Ng-ACP (encoded by Allele 10) proteins showed that the Loop 4 predicted binding interface (Fig 2) for the meningococcal proteins were identical (S4 Fig), but two amino acid changes were observed in Ng-ACP (Nm Val80 to Ng Met80 and Nm Glu81 to Ng Asp81). Thus, to test the hypothesis that the inhibitory function of rNm-ACPI for lysozyme was a general property of *Neisseria* ACP proteins, similar experiments were done with rNm-ACPII (represented by *N*. *meningitidis* strain MC161) and rNg-ACP (represented by *N*. *gonorrhoeae* strain FA1090). The same assay parameters used with rNm-ACPI protein (Fig 4 and Fig 5) were used to examine the dose-dependent kinetics of rNm-ACPII and rNg-ACP inhibition of HL-induced lysis of *M*. *lysodeikticus*. Inhibition of HL activity by rNm-ACPII protein was indistinguishable from rNm-ACPI (S5 Fig and S6 Fig) and the same pattern of lysozyme inhibition *in vitro* is likely to be the case for *N*. *lactamica* ACP protein, which is identical to Nm-ACPI. Kinetic analyses demonstrated that rNg-ACP inhibited HL in an identical manner to rNm-ACP proteins, *i*.*e*. a dose of 0.1 μg/ml rNg-ACP conferred ~30–60% protection of *M*. *lysodeikticus* cells against HL lysis by 2 h (Fig 6A). Higher doses were even more effective, with ≥0.25 μg/ml of rNg-ACP providing ~80–100% protection by 2 h (Fig 6A) and similar levels of inhibition of HL activity by rNg-ACP were observed after 24 h incubation (Fig 6B). Doses of rNg-ACP and rNm-ACPII <0.1 μg/ml were not protective against HL lysis (Fig 6) (P>0.05).

### Modelling of the HL:Nm-ACPI complex

**Fig 7:**
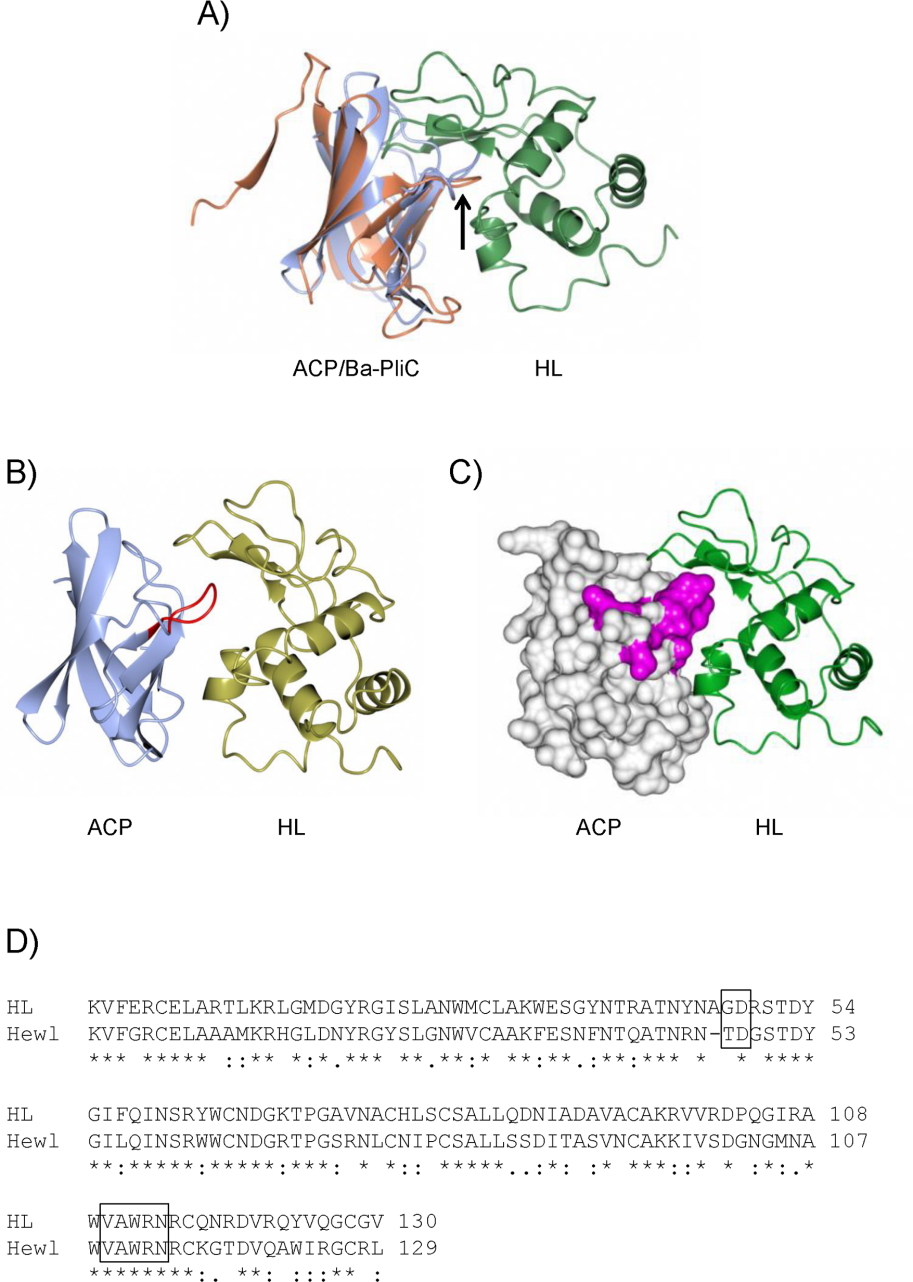
Modelling of the complex between Nm-ACPI and human lysozyme. **A)** Superposition of Nm-ACPI (blue) with the *Brucella abortus* Ba-PliC protein (coral)—HL (green) complex (PDB 4ML7). The black arrow indicates loop 4 from Nm-ACPI/Ba-PliC. **B)** Result of Nm-ACPI/HL docking simulation conducted using RosettaDock; lysozyme is in gold and Nm-ACPI in blue. Loop 4 (residues 76 to 82 inclusive) are highlighted in red. **C)** Surface model of Nm-ACPI (left) in complex with HL, with residues highlighted in magenta that exhibit significant chemical shift changes on binding to Hewl (L74, K76, D78, N79, V80, E81, T82). Images were constructed using CCP4MG [58]. **D)** Sequence alignment of human (HL) and hen egg white lysozyme (Hewl). Residue ranges that interact most closely with ACP in the model with HL are boxed. Identical residues in all sequences in the alignment are marked with (*) at the bottom, conserved and semi-conserved substitutions are marked with (:) and (.) respectively.

Attempts to co-crystallize rNm-ACPI with Hewl or HL were unsuccessful, yielding only crystals of rNm-ACPI alone. Superposition of rNm-ACPI onto coordinates of members of the MliC/PliC family, where structures were available in complex with lysozyme, gave steric clashes which suggested that the mode of binding of rNm-ACPI to lysozyme was different. For example, loop 4 in rNm-ACPI is longer than its equivalent in Ba-PliC, leading to potentially unfavourable interactions with HL (denoted by the arrow in Fig 7A). To obtain a more plausible model of the Nm-ACPI:HL complex, 10,000 independent docking simulations between rNm-ACPI and HL were carried using RosettaDock [28]. The resulting model (Fig 7B) gave a complex in which the HL molecule was significantly further away from ACP than from Ba-PliC, although this could be affected by loop flexibility which is not accounted for by RosettaDock. In this modelled complex, the β4-β8 β-sheet on Nm-ACPI forms an extensive interaction surface with HL, which could inhibit substrate access to the enzyme active site.

To obtain independent experimental evidence to validate the model of the complex, NMR chemical shift mapping was used to identify regions of rNm-ACPI that were perturbed on binding to lysozyme. Using ^1^H,^15^N,^13^C triple resonance experiments, over 80% of the non-proline backbone amide resonances of rNm-ACPI were assigned for the sample conditions used in the experiments. At the high protein concentrations used for NMR data collection, we found that complexes of rNm-ACPI with HL precipitated. The complex of rNm-ACPI with Hewl was, however, soluble and therefore tractable to chemical shift analysis. To identify the lysozyme-binding site(s) on the surface of Nm-ACPI, ^1^H-^15^N HSQC spectra of ^15^N labelled Nm-ACPI alone and in complex with Hewl were compared (S7 Fig). Small but selective chemical shift changes are observed for a number of Nm-ACPI resonances. Most significant are the shift perturbations observed for a contiguous stretch of peptide, spanning residues D74 to T82; when mapped onto the structure of Nm-ACPI, these peptides span loop 4 (Fig 7B and 7C). Shift changes were also observed for isolated amino acids; these were likely due to small differences in sample pH values. Furthermore, sequence alignment of HL and Hewl demonstrated that the residue ranges that interacted most closely with Nm-ACPI in the model with HL were almost identical (Fig 7D). The identification of loop 4 within the β4-β8 region as the principal interaction site between rNm-ACPI and Hewl/HL therefore agrees well with the model proposed from the docking simulations.

### *Neisseria* ACP contributes to HL tolerance *in vivo*

**Fig 8:**
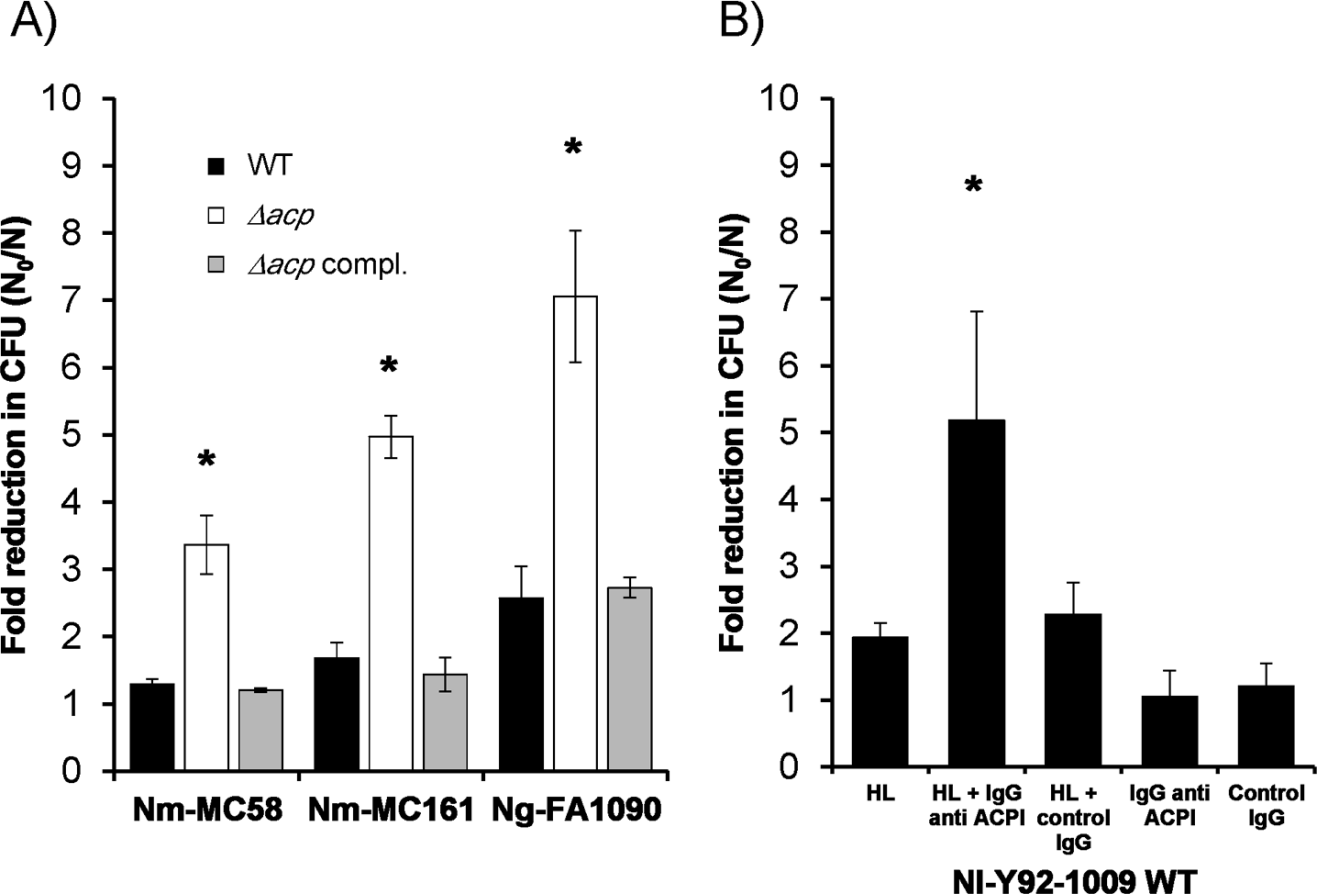
*Neisseria* spp. sensitivity to HL. **A)** Bacterial killing (as measured by the ratio of colony-forming units (CFU) in the absence [N_0_] and presence [N] of human lysozyme: N_0_/N) after 8 h incubation with 10 μg/ml HL and 3 mg/ml lactoferrin, was determined using *N*. *meningitidis* MC58, MC161 and *N*. *gonorrhoeae* FA1090 wild type (black bars), *acp* knock-out (white bars) and complemented (grey bars) strains. **B)** Wild type *N*. *lactamica* sensitivity to HL was addressed by determining the N_0_/N ratio at 8 h after contact with HL and lactoferrin, in the absence or presence of purified IgG antibodies directed against rNm-ACPI (100% identical to NlY92-1009-ACP), or control IgG obtained from pre-immunisation sera. The columns represent the mean and the error bars the standard error of the means calculated from n = 5 independent experiments. Data were compared with a two-sample t-Test, and lysozyme treatments resulting in significant differences (P<0.05) compared to the corresponding controls, are marked with an asterisk (*).

Our data demonstrated that purified *Neisseria* ACP proteins inhibited lysozyme activity using *in vitro* enzyme function assays. We next examined whether expression of ACP by live *Neisseria* spp. was necessary for bacterial survival in the presence of HL. Thus, we compared the growth of *Neisseria acp* knock-out strains (meningococcus MC58Δ*acpI* and MC161Δ*acpII* and gonococcus FA1090Δ*acp*) with the corresponding wild type and complemented strains in the presence of HL. Δ*acp* meningococci and Δ*acp* gonococci showed significant (P<0.05) ~3–5 fold and ~5–9 fold reductions in CFU after 8 h, respectively, whereas wild-type bacteria were relatively insensitive to the effects of HL (Fig 8A; Table 3). Complementation of ACP expression restored resistance to HL at levels similar to wild-type (Fig 8A; Table 3). However, Δ*acp* strains were not completely killed.

**Table 3:** Sensitivity of pathogenic *Neisseria* spp. to HL: Colony Forming Units (CFU) used to plot Fig 8A.

	Bacterium	Colony Forming Units					
	***Neisseria meningitidis***	**WT**		***Δacp***		***Δacp* complemented**	
Expt No.	**MC58**	**- HL (N**_**0**_**)**	**+ HL (N)**	**- HL (N**_**0**_**)**	**+ HL (N)**	**- HL (N**_**0**_**)**	**+ HL (N)**
**1**		870	713	975	278	954	813
**2**		616	505	623	244	575	456
**3**		459	318	528	131	521	442
**Mean ± SEM**		648 ± 120	512 ± 114	709 ± 136	218 ± 44	684 ± 136	570 ± 121
	***Neisseria meningitidis***	**WT**		***Δacp***		***Δacp* complemented**	
**Expt No.**	**MC161**	**- HL (N**_**0**_**)**	**+ HL (N)**	**- HL (N**_**0**_**)**	**+ HL (N)**	**- HL (N**_**0**_**)**	**+ HL (N)**
**1**		525	363	632	133	704	713
**2**		766	525	728	159	795	430
**3**		638	298	705	126	691	467
**Mean ± SEM**		643 ± 70	395 ± 67	688 ± 29	139 ± 10	730 ± 33	537 ± 89
	***Neisseria gonorrhoeae***	**WT**		***Δacp***		***Δacp* complemented**	
**Expt No.**	**FA1090**	**- HL (N**_**0**_**)**	**+ HL (N)**	**- HL (N**_**0**_**)**	**+ HL (N)**	**- HL (N**_**0**_**)**	**+ HL (N)**
**1**		643	183	417	60	409	147
**2**		444	219	390	72	380	156
**3**		844	389	863	98	832	282
**Mean ± SEM**		644 ± 115	264 ± 64	557 ± 153	77 ± 11	540 ± 146	195 ± 44

CFU in the absence [N_0_] and presence [N] of 10 μg/ml human lysozyme and 3 mg/ml lactoferrin after 8 h incubation of *N*. *meningitidis* MC58, MC161 and *N*. *gonorrhoeae* FA1090 wild type (WT), *acp* knock-out (*Δacp*) and complemented (*Δacp* complemented) strains. For each experiment the individual values are the average CFU values from agar plate viable counting used to calculate the mean for n = 3 experiments, with their corresponding standard error of the mean (SEM).

**Fig 9:**
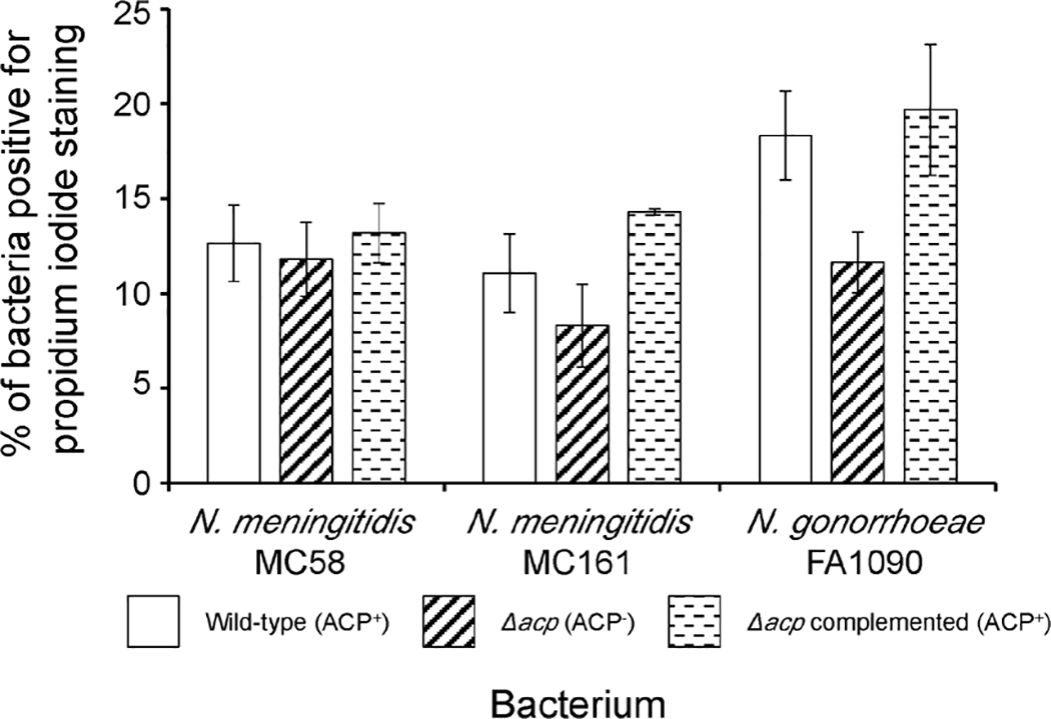
Measurement of membrane permeability using dye viability assay. Wild-type *Neisseria meningitidis* strains MC58 and MC161 and wild-type *Neisseria gonorrhoeae* strain FA1090 and the corresponding *Δacp* and complemented strains were grown overnight on supplemented GC agar medium and colonies were used to inoculate 5 mL of GC broth. Cultures were incubated at 37°C with shaking (200 rpm) until mid-logarithmic growth phase was reached (ODλ_600_nm >0.4) and viability was examined with the LIVE/DEAD *Bac*light Bacterial Viability Kit (Molecular Probes). For each bacterium, approximately 5–6 independent fields of view were examined in duplicate, counting ~100 colonies per field. The percentage of bacteria positive for red propidium iodide (PI) staining, indicating bacteria with permeable membranes, was calculated by dividing PI-positive bacteria by total (red and Syto9 green) bacteria. The columns represent the mean percentages of PI-labelled bacteria (membrane permeant) and the error bars the standard error of the means from a minimum of n = 3 biological replicates per bacterium. A two-sample t-Test was used to compare statistical significance of ACP^-^ viability with ACP^+^ viability data.

We next tested the possibility that the observed increased sensitivity to lysozyme of the Δ*acp* meningococci and Δ*acp* gonococci could have been due to decreased membrane integrity that allowed ingress of extracellular lysozyme. Initially, we quantified the Minimum Inhibitory Concentrations (MIC) of vancomycin and streptomycin antibiotics for wild-type, Δ*acp* and Δ*acp*-complemented meningococci and gonococci (Table 4). Differences in antibiotic susceptibility would be expected if deletion of ACP was causing increased OM permeability. However, there were no significant differences in the MIC values for vancomycin against wild-type, Δ*acp* and Δ*acp*-complemented MC58 and MC161 meningococci (64–96 μg/mL), or for wild-type, Δ*acp* and Δ*acp*-complemented FA1090 gonococci (which were more sensitive than meningococci at 6–8 μg/mL). The MIC values for streptomycin were also similar for the meningococcal wild-type, Δ*acp* and Δ*acp*-complemented variants (16–24 μg/mL), whereas wild-type FA1090 gonococci and the corresponding Δ*acp* and Δ*acp*-complemented variants were relatively insensitive to this antibiotic (MIC>1024 μg/mL). Examination of membrane permeability with the Baclight viability dyes demonstrated that there were no significant differences in the percentages of propidium iodide-labelling of wild-type, Δ*acp* and Δ*acp*-complemented variants of MC58 (11–13%, P>0.05) and MC161 (8–14%, P>0.05) meningococci or FA1090 gonococci (11–19%, P>0.05) (Fig 9).

**Table 4:** Minimum inhibitory concentration (MIC) of vancomycin and streptomycin antibiotics for wild-type, *Δacp and* *Δacp* complemented meningococci and gonococci.

Antibiotic	Bacterium										
	*Neisseria meningitidis* MC58			*Neisseria meningitidis* MC161				*Neisseria gonorrhoeae* FA1090			
	WT	*Δacp*	*Δacp* compl.		WT	*Δacp*	*Δacp* compl.		WT	*Δacp*	*Δacp* compl.
Vancomycin MIC (μg/mL)	96	96	96		64–96	64–96	48–64		6–8	8	8
Streptomycin MIC (μg/mL)	16	16	16–24		16	16	16		>1024	>1024	>1024

Lawns of *N*. *meningitidis* MC58, MC161 and *N*. *gonorrhoeae* FA1090 wild type (WT), *acp* knock-out (*Δacp*) and complemented (*Δacp* compl.) strains on solid GC supplemented agar plates were exposed to Etest Vancomycin and Streptomycin strips, with overnight incubation at 37°C with 5% (v/v) CO_2_. MIC values were determined as described by the manufacturer and the values represent the mean determinations from at least two independent biological replicates.

To our knowledge there are no published general protocols available for generating *N*. *lactamica* gene knock-out mutants. A method has been presented at a conference that reported successful transformation of a kanamycin resistance gene into *N*. *lactamica*, with the use of hypermethylated (*i*.*e*. restriction resistant) PCR products as donor material leading to a 1000-fold increase in transformation efficiency [29]. Nevertheless, as robust and reproducible mutagenesis methods are not generally available, we used an alternative strategy to investigate the role of ACP for survival of *N*. *lactamica* commensal organism in the presence of HL. An assay was developed in which purified anti-Nm-ACPI rabbit IgG was reacted with live wild-type *N*. *lactamica* strain Y92-1009 in order to block ACP function. In the presence of this specific antibody and HL, we observed a significant ~3–8 fold reduction (P<0.05) in *N*. *lactamica* CFU over an 8h incubation. In general, the *N*. *lactamica* Y92-1009 wild type strain was relatively insensitive to HL, similar to wild-type meningococci and gonococci (Fig 8B; Table 5). Addition of pre-immunization rabbit IgG with and without HL or anti-Nm-ACPI rabbit IgG alone did not affect wild-type bacterial growth.

**Table 5:** Sensitivity of *N*. *lactamica* to HL: Colony Forming Units (CFU) used to plot Fig 8B.

	*Neisseria lactamica* Y92-1009 WT Colony Forming Units						
Expt No		- HL (N_0_)	+ HL (N)	HL + IgG anti-ACPI (N)	HL + control IgG (N)	IgG anti-ACPI (N)	Control IgG (N)
**1**		75	33	17	29	137	72
**2**		58	30	18	25	73	80
**3**		60	44	22	63	Nd	Nd
**4**		151	69	26	64	83	81
**5**		153	77	16	48	Nd	Nd
**Mean ± SEM**		99 ± 22	51 ± 10	20 ± 2	46 ± 8	98 ± 15	78 ± 2

CFU of *N*. *lactamica* Y92-1009 WT in the absence [N_0_] and presence [N] of 10 μg/ml human lysozyme and 3 mg/ml lactoferrin, with or without the addition of purified IgG antibodies directed against rNm-ACPI (100% identical to NlY92-1009-ACP), or control IgG obtained from pre-immunisation sera [N]. For each experiment the individual values are the average CFU values from agar plate viable counting used to calculate the mean for n = 5 experiments, with their corresponding standard error of the mean (SEM). Nd, not determined.

## Discussion

In the current study, our data suggest that *Neisseria* ACP, a surface-exposed OM molecule with a role in mediating meningococcal adhesion and capable of inducing a functional bactericidal antibody response [20], is involved also in inhibiting C-type human lysozyme activity. To the best of our knowledge, this represents the first report of ACP functioning as a novel lysozyme inhibitor for both pathogenic and commensal *Neisseria* spp. Moreover, examination of ACP allelic variation amongst 12,483 *Neisseria* isolates (http://pubmlst.org/neisseria/, analysed on December 2016) shows that there is a high degree of conservation of this lysozyme inhibitor. There are 153 different *acp* alleles encoding 43 non-redundant ACP protein sequences (S1 Table): Allele 2 is expressed by the largest number of isolates that are almost exclusively *N*. *meningitidis* strains (7966/8568 (93%) of meningococcal isolates and 7966/12483 (64%) of all *Neisseria* spp. isolates). The second most commonly expressed ACP protein by *N*. *meningitidis* is encoded by Allele 1 (570/8568, representing 7% of meningococcal isolates), which is also the major ACP protein expressed by commensal *N*. *lactamica* (100/139, representing 72% of all *N*. *lactamica* isolates). Allele 10 and Allele 6 are expressed predominantly by *N*. *gonorrhoeae* strains (3021/3737 (81%) of gonococcal isolates and 3021/12483 (24%) of total *Neisseria* spp. isolates for Allele 10; and 553/3737 (15%) of gonococcal isolates and 553/12483 (4%) of total *Neisseria* spp. isolates for Allele 6) (S1 Table). Alignment of all 43 non-redundant *Neisseria* ACP protein sequences shows a high degree of amino acid sequence conservation within the mature proteins (amino acids 22–124) (S8 Fig). Notably, *Neisseria* ACP amino acid sequences encoded by Alleles 1 and 2 show ≥99% identity and there is only one single amino acid substitution between Type I (Asp25) and Type II (Asn25) proteins (S8 Fig). Alleles 6 and 10 encoded gonococcal ACP proteins are 98% identical, with the same single amino acid substitution (Asp25 to Asn25) and a deletion of Ala22 in Ng-ACP protein encoded by Allele 10 (S8 Fig). Thus, the four major proteins encoded by *acp* Alleles 1, 2, 6 and 10 show ~94% amino acid sequence similarity and cover 98% of *Neisseria* isolates in the http://pubmlst.org/neisseria/ database.

To our knowledge, the current study is the first to determine the structure of the *Neisseria* ACP protein by crystallography and suggests potential differences in the mechanism of lysozyme inhibition compared with known lysozyme inhibitor families found in other Gram-negative bacteria. These families have been classified according to substrate specificity, the presence of specific sequence motifs and structural topology, *i*.*e*.—Ivy from *E*.*coli* [10], MliC/PliC from *S*. *enteritidis*, *E*. *coli* and *P*. *aeruginosa* [11, 24], PliI from *Aeromonas hydrophila* [30], PliG from *E*. *coli* [31] and the recently discovered PliG-type Tsi3 from *P*. *aeruginosa* [12, 32]. Bacterial lysozyme inhibitors have conserved motifs within the C-terminal region, which are located within protruding loops involved in enzyme inhibition. Previous structural studies for MliC/PliC family lysozyme inhibitors, *e*.*g*. *P*. *aeruginosa* MliC in complex with Hewl (PDB 3F6Z) [24], *E*. *coli* MliC [33] and *S*. *typhimurium* PliC (PDB 3OE3) [22] have shown that they all fold into eight-stranded anti-parallel β-barrels and share two conserved sequence motifs. The first conserved region (Sx**S**GAx**Y**), present in the MliC/PliC, PliI and PliG family proteins, is mainly located on the fourth loop between β4-β5 strands: the second conserved region (Yxxx**TK**G) is located on the sixth β-strand of proteins and is only found in the MliC/PliC family [1]. Structural data supported by mutational analysis has provided insight into the molecular mechanism by which these proteins interact with and inhibit lysozyme, and a ‘double key-lock’ mechanism has been suggested [24].

In our current study, the Nm-ACPI crystal structure, has demonstrated that *Neisseria* ACP proteins are structurally similar to the MliC/PliC family, but do not share primary sequence similarity nor any described sequence motifs with this family [11], or with any other characterized bacterial lysozyme inhibitor reported to date [1]. Although a central role for the *Neisseria* ACP loop 4 is suggested for binding to and inhibition of lysozyme, this loop is three amino acid residues longer than the MliC/PliC loop 4. A structural model of Nm-ACPI in complex with HL, obtained by docking simulation, showed loop 4 is predicted to insert into the HL active cleft, and the β4-β8 β-sheet on Nm-ACPI forms an extensive interaction surface with lysozyme, which could inhibit substrate access to the enzyme active site. NMR-specific chemical shift changes of Nm-ACPI in the presence of HL supported our proposed mode of interaction. These observations, together with the fact that *Neisseria* ACP proteins cannot be classified into any of the known and well-characterised lysozyme inhibitor families due to the lack of significant primary sequence homology and absence of conserved specific sequence motifs, support our hypothesis that this protein is a novel lysozyme inhibitor that binds to and inhibits lysozyme via different sequence motif(s). More generally, our study supports the notion that lysozyme inhibitors in Gram negative bacteria are more diverse than previously thought. For example, recent studies have shown that there are mechanistic differences in lysozyme inhibition, even between members of the same family, *e*.*g*. the identification of a new binding interface in *Brucella abortus* PliC complexed with HL, which was disordered in *P*. *aeruginosa* MliC complexed with Hewl [23]. In addition, for lysozyme inhibitors belonging to the PliI or PliG family, only the first motif loop is conserved and surface-exposed and predicted to inhibit lysozyme by inserting into the active-site cleft [22].

To try to confirm our model of the complex, we attempted mutagenesis of a number of sites within ACP and characterized a panel of single mutants. However, there was no single site mutation that had a significant effect on ACP activity, although a triple mutant (Asn79Ala/Tyr84Ala/Gly95Ala) showed ~39% reduced activity compared with the wild-type (S2 Table). Since mutagenesis of individual sites had little effect and the observation with a triple mutant is insufficient on its own to validate the model, we hypothesise that the energetics of binding are distributed across several different residues. Instead, the NMR data provide much stronger validation for the model.

Our study has shown that ACP inhibits death by lysozyme of *Micrococcus* and *Neisseria* spp. In addition to its muramidase activity, it is known that lysozyme also displays antimicrobial peptide (AMP) activity. However, several lines of evidence support our conclusion that *Neisseria* ACP inhibits peptidoglycan degradation by lysozyme. First of all, we demonstrated conclusively by MST and NMR that *Neisseria* ACP bound to lysozyme. In particular, the NMR data are a good test of specificity, with observed chemical shift changes for a limited range of residues, which is characteristic of specific binding. By contrast, non-specific binding would have been manifested by chemical shift changes by many different surface residues, as the lysozyme would have multiple binding sites on ACP. Secondly, we confirmed that boiled lysozyme did not affect *Micrococcus* integrity [34], indicating that lysozyme antimicrobial activity was not responsible for the observed *Micrococcus* cell death in our *in vitro* assays. Moreover, lysozymal AMP activity, in common with other AMPs, does not degrade peptidoglycan directly [35, 36]. Thirdly, direct peptidoglycan degradation by lysozyme is reported with the *in vitro* assay using fluorescein-labelled cell walls of *Micrococcus*. In this assay, cell wall peptidoglycan-fluorescein is the lysozyme substrate and increase in fluorescence intensity is due to the enzymatic activity of lysozyme and not AMP activity. Thus, the observed reduction in fluorescence in the presence of *Neisseria* ACP was attributable to the capacity of ACP to inhibit peptidoglycan degradation by lysozyme. In addition, we demonstrated that deletion of *acp* gene was associated with increased sensitivity of *Neisseria* spp. to lysozyme *in vivo*, without affecting bacterial membrane integrity or permeability. Taken together, these lines of evidence support our conclusion that ACP inhibits lysozymal degradation of peptidoglycan.

We have shown also that meningococcal ACP proteins are OM-located and surface-exposed [20] (S3 Fig). By contrast, other Gram-negative bacteria lysozyme inhibitor proteins are present either in the periplasm or anchored to the luminal face of the OM. *Neisseria* ACP proteins do not possess a recognisable lipobox and are unlikely to be anchored to the OM in a lipidated form, unlike membrane-bound MliC family proteins such as *P*. *aeruginosa*-MliC and *Mycobacterium tuberculosis* lipoprotein LprI [13]. Interestingly, the face associated with the N-terminal region of *Neisseria* ACP and opposite to the predicted HL-binding site, is highly positively charged (Fig 1 and Fig 7), which could provide ACP with the ability to associate with negatively charged surfaces and possibly mediate attachment to the membrane or capsule surface. Electrostatic binding interactions have been reported for other bacterial proteins, *e*.*g*. *E coli* protein antibiotic colicin N [37] and *P*. *aeruginosa* OprH [38] both interact with lipo-oligosaccharide chains, and *P*. *aeruginosa* G-type lysozyme Tse3 interacts with the inner leaflet of the OM [32].

The ability of ACP proteins to inhibit lysozyme function clearly provides *Neisseria* spp. with the potential to avoid a key vertebrate innate immune defence mechanism found on all mucosal surfaces and secretions [2, 3] and in the respiratory airway [4]. Our study shows that ACP proteins conferred increased lysozyme tolerance to both pathogenic and commensal *Neisseria* spp. and showed higher activity against human lysozyme compared to avian lysozyme. A hypothetical model can be proposed to describe how expression of ACP enables *Neisseria* spp. interaction with the host. *Neisseria* ACP protein(s) functions as an adhesin primarily for epithelial cells [20] and is likely to contribute to mechanisms of adherence of meningococci and commensal *Neisseria* spp. to nasopharyngeal mucosal epithelium and gonococci to urogenital mucosal epithelia. Evasion of bacteriolysis during host colonization by *Neisseria* spp. is likely to be effected by multiple and possibly non-redundant mechanisms. Thus, a combination of PG acetylation [14, 15], ACP expression and other recently described mechanisms such as expression of LtgA and LtgD lytic transglycosylases [34], enables both pathogenic and commensal *Neisseria* species to limit human innate immune clearance by lysozyme and contribute to establishing colonization. C-type lysozyme is present also in blood [7] and for potentially invasive species such as meningococci, it is possible that expression of ACP may be important for survival.

In summary, we have identified a new function for *Neisseria* ACP as an OM-located, surface-exposed lysozyme inhibitor, in addition to its role as an adhesin and as a potential vaccine candidate. ACP is found in all pathogenic and commensal *Neisseria* spp., and despite structural similarity to the MliC/PliC family proteins, it shares no conserved motifs involved in lysozyme inhibition. Thus, ACP could represent a distinctive protein different to the known families of bacterial lysozyme inhibitors [1]. Our data suggest that the ability of ACP to inhibit lysozyme activity could be important for host colonization, not only by pathogenic meningococci and gonococci, but also by commensal organisms. Thus, ACP represents a dual target for developing *Neisseria* vaccines and drugs to inhibit host-pathogen interactions.

## Materials and methods

### Bacteria, growth conditions and preparation of outer membranes (OM)

*Neisseria meningitidis* strains MC58 (B: 15:P1.7, 16b) and MC161 (C: 2–37, P1.5–1, 10–4) have been described previously [39, 40]. *Neisseria gonorrhoeae* strain FA1090 was purchased from the American Type Culture Collection (ATCC 700825) and *Neisseria lactamica* strain Y92-1009 (sequence type 3493, clonal complex [CC] 613) was produced by the Current Good Manufacturing Practices pharmaceutical manufacturing facilities at Public Health England (Porton Down, United Kingdom). Wild type, mutant and complemented strains were grown on supplemented GC agar plates (with the addition of selective antibiotics and/or isopropyl-β-1-D-thiogalactopyranoside (IPTG; Sigma-Aldrich) when necessary) and incubated at 37°C with an atmosphere of 5% (v/v) CO_2_ [41]. *Escherichia coli* DH5α or XL-10 Gold ultra-competent *E*. *coli* cells (Stratagene) (cloning) and BL21(DE3) pLysS (New England Biolabs) (protein expression) strains (Invitrogen, UK) were grown on Luria-Bertani (LB) agar, LB, SOB or Terrific Broth with or without addition of selective antibiotics and IPTG when necessary.

Whole suspensions of MC58 and MC161 were prepared in water and OM were prepared by extraction of whole bacteria with 0.2 M lithium acetate buffer, pH 5.8 and differential ultracentrifugation, as described previously [42, 43].

### Construction of *Neisseria meningitidis nm-acp* gene and *Neisseria gonorrhoeae ng-acp* gene knock-out mutants

Construction of MC58Δ*acpI* mutant strain has been described previously [20] and construction of MC161Δ*acpII* mutant strain was done using the same protocol. Briefly, primers KO2095F (5’-CGGGCTGAACCAGATAGACT-3’) and KO2095R (5’-GCTCCAGTTTGGTACGGAGA-3’) were used to amplify the 2.9 kb DNA segment from the genomic DNA of the *acp*^*-*^ mutant 35/11 [44]. In this strain, the *nm-acp* ORF was interrupted by the insertion of a mini-transposon (1.6 kb) [44], while the amplified PCR product from the wild-type MC58 or MC161 strains was 1.3 kb. The 2.9 kb PCR product was gel purified, cleaned, and used for transformation of naturally competent MC161 strain [45]. Transformed bacteria were screened by PCR, and the selected MC161Δ*acpII* colonies were confirmed as Nm-ACP^-^ by Western blot analysis using murine anti-Nm-ACP sera.

To generate a Δ*acp* isogenic deletion mutant in gonococcal strain FA1090, the target gene was replaced by a kanamycin (Kan) resistance cassette (following the method described by Echenique-Rivera *et al*. [46], with modifications). Approximately 400-bp fragments of the flanking regions of the target gene were amplified by PCR from FA1090 genomic DNA. A 400-bp fragment upstream of *ng-acp* (F1) was generated using a forward primer (F1-Fw; 5’-TAGACTTCTGGGGCAAGGTC-3’) and a reverse primer (F1-Rv; 5’-GGCTATTCTAGATTTTATTCCTTTGGATAGATG-3’), carrying the restriction site for XbaI (underlined). A 400-bp fragment downstream of the target gene (F2) was amplified using a forward primer (F2-Fw; 5’GGCTATTCTAGATCAGGCAACAAAAAACAGCG-3’), also incorporating a restriction site for XbaI, and a reverse primer (F2-Rv; 5’-GGTACGGAGATTGTCGCCC-3’). The PCR products were purified using a Wizard SV Gel and PCR Clean-up System (Promega), digested with XbaI restriction enzyme (Promega) at 37°C for 3 h, and purified from an agarose gel band. The purified digested PCR fragments were then ligated overnight at 4°C and used as a template for amplification by PCR using F1-Fw and F2-Rv primers. The ~800-bp PCR product was purified and cloned into the pGEM-T Easy vector (Promega), which was then used to transform competent *E*. *coli* DH5α cells. Purification of the construct (pGEM::F1+F2) was performed by mini-prep from a positive transformed colony, and the plasmid was then digested with XbaI in order to insert a Kan cassette in between fragments F1 and F2. The kanamycin resistance cassette was previously amplified by PCR with primers KanFw (5’-GGTTCTAGA*TTCAGACGGC*GTGATCTGATCCTTCAACTC-3’) and KanRv (5’- GGTTCTAGATTAGAAAAACTCATCGAGCATC-3’), which incorporated an XbaI restriction site and a DNA uptake sequence (in *italics*). The PCR product was purified, digested with XbaI and purified from an agarose gel band before ligation to linearized pGEM::F1+F2 (o/n at 4°C). This ligation product (pGEM::F1+Kan+F2) was used to transform *E*. *coli* DH5α cells and subsequently the naturally competent FA1090 strain. Mutagenesis was achieved by heterologous allelic exchange. Transformed colonies were screened by PCR, and the selected FA1090Δ*ng*-*acp* strain was confirmed by Western blotting using cross-reacting murine anti-Nm-ACP sera.

### Complementation of meningococcal and gonococcal Δ*nm/ng-acp* strains

Complementation of MC161Δ*nm-acpII* and FA1090Δ*ng-acp* knock-out strains was performed as previously described for the complementation of MC58Δ*nm-acpI* mutant strain [20]. Primers Com2095F (5’-GGCTATTTAATTAAATGAAACTTCTGACCACCGC-3’) and Com2095R (5’- TTAACGTGGGGAACAGTCTT-3’) were used to amplify *nm-acpII* and *ng-acp* genes from MC161 or FA1090 genomic DNA respectively, cloned into pGCC4 vector in between the PacI and PmeI restriction sites (under transcriptional control of a *lac* promoter) and confirmed by sequencing using an upstream primer, LacPFw (5’-CGGTTCTGGCAAATATTCTG-3’). The resulting constructs pGCC4-*nm-acpII* and pGCC4-*ng-acp* were transformed into MC161Δ*nm-acpII* or FA1090Δ*ng-acp* knock-out strains respectively using the method of Stohl and Seifert [45], and the complementary strains were identified by PCR screening and Western blotting with murine anti-Nm-ACP sera.

### Cloning, expression, and purification of recombinant *Neisseria* ACP proteins

The *nm-acpI and II* gene sequences, optimized for *E*. *coli* expression and encoding the entire coding sequence for NMB2095 protein (NEIS2075, http://pubmlst.org/neisseria/, 375 bp), were synthesized *in vitro* (GeneArt, Invitrogen). Primers gACP-Fw (5’- GGCTATCATATGAAACTTCTGACCAC -3’) and gACP-Rv (5’- GGCTATCTCGAGACGTGGGGAACAG -3’) were used to amplify the complete NEIS2075 ORF from *N*. *gonorrhoeae* strain FA1090 genomic DNA. All *acp* genes were cloned into the pET22b(+) system (Novagen) in between the NdeI and XhoI restriction sites providing a C-terminal hexa-histidine tag. Recombinant plasmid (pET-*nm-acpI*, pET-*nm-acpII* and pET-*ng-acp*) was transformed into either *E*. *coli* XL-10 Gold ultra-competent or *E*.*coli* DH5α cells for plasmid amplification and subsequently into competent *E*. *coli* BL21 (DE3) pLysS cells for protein expression.

In order to produce ultra-high purity recombinant (r)Nm-ACPI for X-ray crystallography, *E*. *coli* BL21 (DE3) pLysS carrying plasmid pET-*nm-acpI* was grown in Terrific Broth and induced at an optical density (OD) λ_600_nm of 0.8 by adding IPTG to a final concentration of 0.5 mM and left to grow for 12–14 h at 16 ^0^C. To produce ^15^N- and ^13^C/^15^N-labelled rNm-ACPI for NMR studies, *E*. *coli* cells were grown in M9 minimal media (6 g/l Na_2_HPO_4_, 3 g/l KH_2_PO_4_ pH 7.4, 0.5 g/l NaCl, 1 g/l ^15^NH_4_Cl, 0.2 mM/l CaCl_2_, 1 mM/l MgSO_4_ and 20 ml of 20% ^13^C Glucose) supplemented with 200 mg/l ^15^N- or ^15^N ^13^C Isogro media (Sigma-Aldrich). Stable isotope-labelled rNm-ACPI was subsequently purified following the same procedure as for the unlabelled protein (see below). Purified protein was dialysed into 50 mM sodium phosphate buffer pH 6.0 for NMR spectroscopy. Bacteria were harvested by centrifugation at 10,000 x *g*_*av*_ for 20 min at 4 ^0^C and subsequently suspended in lysis buffer (50 mM HEPES/NaOH buffer, pH 7.5, 200 mM NaCl, 5% v/v glycerol) supplemented with 0.2 mg DNase (Sigma) and 1x EDTA-free protease inhibitor cocktail (complete, Roche). Bacteria were broken using a probe sonicator set at 30% amplitude for 5 min with 5 s ON and 10 s OFF pulses to release soluble rACP proteins, which were recovered from the supernatant fraction after centrifugation at 27,000 x *g*_*av*_ for 30 min at 4 ^0^C. The supernatant was passed through a 0.45 μm filter then stored at 4 ^0^C with 2 ml Ni-IDA (nickel iminodiacetic acid) resin (Generon) for 1 hr with gentle mixing. After storage, the mixture was passed through a gravity flow column to pack the rACP-bound resin. Unbound proteins were washed with 20 column volumes of wash buffer (50 mM HEPES/NaOH buffer, pH 7.5, 200 mM NaCl, 40 mM imidazole, 5% v/v glycerol), while bound rNm-ACPI was eluted with increasing concentrations of imidazole up to 500 mM in wash buffer. Protein samples obtained were analysed using 12% (w/v) SDS-PAGE (Invitrogen). rNm-ACPI was subsequently dialysed against 25 mM HEPES/NaOH buffer, pH 7.5 at 4 ^0^C for 12–14 h and was purified further by ion exchange chromatography with a cation exchanger, Resource S column (GE Healthcare) using a linear gradient from dialysis buffer A to buffer B (25 mM HEPES/NaOH, pH 7.5, 1 M NaCl). Eluted rNm-ACPI was concentrated with 5 kDa cut-off Vivaspin centrifugal concentrators (Sartorius) at 6,000 x g_av_ then further purified with a size exclusion chromatography column, Superdex S-75 Hi-Load 16/600 column (GE Healthcare) on an AKTA-FPLC system (GE Healthcare) using 25 mM HEPES/NaOH buffer, pH 7.5 and 150 mM NaCl as running buffer. Eluted protein was analysed for purity by SDS-PAGE (S1 Fig), and concentrated. Protein concentration was determined at 1600–1690 cm^-1^ wave number using a Direct Detect FTIR spectrometer (EMD Millipore).

Protein identification of purified rNm-ACPI for crystallography was confirmed by mass spectrometry of excised SDS-PAGE bands. Excised gel bands were dehydrated using acetonitrile and subsequently trypsin digested overnight at 37°C. LC-MS/MS was done using an UltiMate 3000 Rapid Separation Liquid Chromatography system (Dionex) coupled to a LTQ Velos Pro (Thermo Fisher Scientific) mass spectrometer. Peptide mixtures were separated using a gradient from 92% A (0.1% formic acid in water) and 8% B (0.1% formic acid in acetonitrile) to 33% B, in 44 min at 300 nL min^-1^, using a 250 mm x 75 μm i.d. 1.7 mM BEH C18, analytical column (Waters). Peptides were selected for fragmentation automatically by data dependant analysis. Using Mascot (Matrix Science), data produced were searched against the UNIPROT database with taxonomy of *Neisseria* spp. and were subsequently validated using Scaffold (Proteome Software).

rNm-ACPII and rNg-ACP proteins were purified also using Ni-IDA affinity chromatography under native conditions, but bound proteins were eluted using 50 mM NaH_2_PO_4_, 300 mM NaCl, 250 mM imidazole buffer, pH 8.0, and subsequently dialyzed against PBS, pH 7.4 for 48 h. Protein concentration was determined using the BCA Protein Assay Kit (Pierce). The molecular mass (*Mr*) of mature rNm-ACPI, rNm-ACPII and rNg-ACP proteins, without their leader peptide sequence and each with a C-terminal hexa-histidine tag, was ~12.3 kDa, as shown by SDS-PAGE (S1 Fig). All recombinant proteins were stored at -80 ^0^C until needed.

### Crystallization and structure determination of rNm-ACPI

Crystallization was performed by the sitting drop method using MRC 96 Well 2 Drop Crystallization Plates (Molecular Dimensions) using a Mosquito crystallization robot (TTP Laptech). Wild type rNm-ACPI crystals were grown at 20 ^0^C by mixing 200 nl of protein solution (20 mg/ml in 25 mM HEPES/Na pH 7.5, 37 mM NaCl) with 200 nl precipitant solution containing 0.2 M lithium sulfate, 0.1 M sodium acetate pH 4.5 and 50% (w/v) PEG 400 (JCSG plus, Molecular Dimensions), with a reservoir volume of 50 μl. Crystals were cryoprotected by washing for 5 min in well solution supplemented with 20% (v/v) glycerol, before flash cooling in liquid nitrogen. For phasing, crystals were incubated for 10 min in the well solution supplemented with freshly prepared 0.4 M KI, which was also included in the cryoprotectant. Diffraction data were collected at Diamond Light Source beamlines I04-1 and I03. Datasets were processed by automated pipeline implemented in xia2 [47], using XDS [48]; data collection statistics for native and KI-soaked crystals are summarised in Table 1. Reflection data from native and KI-soaked crystals, were merged and scaled using Aimless [49], as implemented in the CCP4 suite [50]. For phasing, automated heavy atom substructure identification was combined with experimental phasing and model building using Autosol [51], as implemented in PHENIX [52]. Autosol identified and refined the locations of 8 iodine sites (figure of merit 0.289); following density modification by RESOLVE, a readily interpretable electron density map was produced. An initial model was produced by Autobuild [53], which built the majority of the residues. The structure was completed by manual model building in Coot [54], and refined using REFMAC [55] and PDB_REDO [56]. The relevant phasing statistics and refinement parameters are detailed in Table 2.

### Microscale thermophoresis (MST)

Biomolecular interaction between rNm-ACPI and HL (Sigma-Aldrich) was studied using the Monolith NT.115 microscale thermophoresis instrument, (NanoTemper Technologies GmbH) and a light-emitting diode (LED) filter of 605–645 nm excitation and 560–685 nm emission. The rNm-ACPI protein (2.5 mM) was dialysed overnight at 4 ^0^C into MST buffer (50 mM Tris pH 7.4, 150 mM NaCl, 10 mM MgCl_2_ and 0.05% (v/v) Tween-20). Recombinant (r)HL (20 μM) was labelled using the Monolith NT Protein Labeling Kit (NanoTemper Technologies) according to the manufacturer’s protocol. In brief, 100 μl of 20 μM rHL was incubated at room temperature with 100 μl of 60 μM NT-647-NHS fluorescent dye (NanoTemper Technologies) reconstituted in DMSO. Labelled rHL protein (3.33 μM) was recovered with 600 μl of MST buffer using a buffer exchange column.

The binding affinity measurements between rNm-ACPI and rHL were carried out as described elsewhere [57]. Two fold serial dilutions up to 16 dilutions of 2.5 mM rNm-ACPI were prepared in MST buffer and mixed in a 1:1 ratio with 8.3 nM NT647-labelled rHL to yield a final volume of 20 μl per dilution. The reaction mixtures were loaded into premium-coated capillary tubes (NanoTemper Technologies). The interaction was analysed by MST at 20% and 40% MST power and a light-emitting diode (LED) intensity of 20%. Interaction by thermophoresis was analysed after 20 s laser-on time and dissociation constants (K_d_) were calculated from concentration dependent changes in normalised fluorescence (F_norm_) of NT647-HL after 5 s of thermophoresis using the NTAffinity Analysis software. Experiments were carried out in triplicate and arithmetic means and standard deviations of readings used.

### *In silico* docking of Nm-ACPI onto HL

The structure of Nm-ACPI was superposed using CCP4MG [58] onto PliC from *Brucella abortus* in the complex with HL (PDB accession code 4ML7). This modelled complex of Nm-ACPI was used as the starting point for 10,000 independent docking simulations carried out using RosettaDock [28]. The complex with the lowest energy was selected.

### NMR spectroscopy

NMR spectra were recorded at 25°C on a Bruker DRX 800 spectrometer equipped with CryoProbes. Sequence-specific backbone resonance assignment of rNm-ACPI was obtained using multi-dimensional heteronuclear NMR experiments HNCACB and CBCA(CO)NH. ^15^N-rNm-ACPI in complex with Hewl was prepared by mixing ^15^N-rNm-ACPI with unlabelled Hewl followed by SEC purification. For comparison of the ^15^N-^1^H HSQC spectra obtained from the free rNm-ACPI and in complex with Hewl, the data were collected from the same batch of ^15^N-labelled rNm-ACPI, in order to minimise any differences in conditions between the free and complexed protein. Data were processed using the Bruker Software TopSpin and analysed using CCPN software [59].

### Generation of murine antisera to mature rNm-ACPI and rNm-ACPII proteins

BALB/c mice (H-2^d^ haplotype) were bred within the animal facilities of the university under standard conditions of temperature and humidity with a 12 h lighting cycle and with food and water available *ad libitum*. Groups of five BALB/c mice of approximate equal sizes and weights (6–7 weeks of age) were immunized intraperitoneally with purified mature rNm-ACP proteins (Types I and II) in saline solution, liposomes or Al(OH)_3_ formulations, prepared using methods described previously [20]. Within each group, individual mice were immunized with 20 μg of recombinant protein on days 0, 14, and 28. Groups of five mice were also sham immunized (no protein) and one group was kept for normal mouse serum (NMS). Mice were terminally bled by cardiac puncture under anesthesia on day 42. All sera were stored at −20°C until required and decomplemented by heating at 56°C in a water bath for 30 min before use.

The quality and specificity of murine anti-rNm-ACPI and anti-rNm-ACPII sera were assessed by i) Enzyme-Linked ImmunoSorbent assay (ELISA) reactivity against recombinant protein and MC58 and MC161 OM preparations and ii) Western immunoblotting on wild type MC58 and MC161 OM preparations as described previously [60] (S3 Fig). Binding of antibodies to bacterial surfaces was demonstrated by Fluorescence-Activated Cell Sorter (FACS) analysis, as described previously [20] (S3 Fig).

### Ethics statement

This study complied with the animal experimentation guidelines of the Home Office (HO), with approval granted under the Animals (Scientific Procedures Act, 1986) with a HO project licence number PPL 30/3126. The study was approved by the Animal Welfare and Ethics Review Board (AWERB) at the authors’ institution (University of Southampton, no number assigned). Animal health and welfare was assessed daily by qualified animal technicians and no animals suffered significant adverse effects.

### Purification of anti-rNm-ACPI IgG antibodies

Polyclonal IgG antibodies were purified from rabbit antisera that were generated to rNm-ACPI in our previous study [20], using the Pierce Fab Preparation Kit, following the manufacturer’s protocol with some variations. Briefly, rabbit pre- and post-Nm-ACPI immunisation sera were desalted with a Zeba Spin Desalting Column and incubated for 10 min in a Nab Protein A Plus Spin Column at room temperature. Purified IgG antibodies were recovered with Elution buffer pH 2.8 (which contains primary amine) and immediately neutralized with 1M Tris buffer, pH 8.5. Elution samples were then concentrated and dialyzed against PBS, pH 7.4 with Amicon Ultra-4 centrifugal filters (Merck Millipore). Protein concentration was finally determined by absorbance at λ_280_nm using an estimated extinction coefficient of 1.4 and finally analysed by non-reducing and non-boiled SDS-PAGE.

### Lysozyme inhibition assays

#### Hen egg-white lysozyme (Hewl) inhibition assay

Hewl inhibition mediated by rNm-ACPI protein was studied *in-vitro* using the EnzChek Lysozyme assay kit (Molecular Probes, Life Technologies) that contains a *Micrococcus lysodeikticus* suspension in which the bacterial cell walls are labelled with fluorescein to such an extent that the fluorescence is quenched. The suspension acts as a DQ lysozyme substrate, such that release of the quenched fluorophore upon hydrolysis of PG by lysozyme action gives higher fluorescence signal intensities [61]. Each assay consisted of 100 μl volumes of 0.1 M sodium phosphate, 0.1 M NaCl, pH 7.5 and 2 mM sodium azide, containing 10 U of Hewl incubated with rNm-ACPI (0.2–0.00156 mg) in the test wells and either 10 U Hewl or 0.2 mg of rNm-ACPI in the control wells. Replacement of rNm-ACPI with an heterologous recombinant protein from *Neisseria meningitidis*, Macrophage Infectivity Potentiator (rNm-MIP) served as a negative control. The reaction was initiated by the addition of 50 μg of fluorescein-labelled substrate. Fluorescence was measured every 10 min over 2 h at 37 ^0^C with 5 s of plate shaking before each reading using a Synergy HTX Multi-Mode Microplate Reader (BioTek) at excitation and emission wavelengths of 485 nm and 530 nm respectively. Results were compiled and analysed using BioTek Gen5 software via a Microsoft Excel interface. The fluorescence intensity for each reaction well was normalised using values derived from substrate only controls. Two human single heavy chain variable V_H_ domain antibody fragments, one of which—HEL4 V_H_-Ab—has been shown to bind to lysozyme but not at the active site [62] and the other—H04 V_H_-Ab—which binds and occupies the active site of lysozyme [63], were also included in the assay as negative and positive controls, respectively.

#### Human neutrophil lysozyme (HL) inhibition assay

A lysozyme inhibition assay was done using freeze-dried *M*. *lysodeikticus* cells (ATCC4698 –Sigma Aldrich) suspended at 1 mg/ml in 10 mM potassium phosphate buffer (PPB) pH 7.0 supplemented with a protease inhibition cocktail (Roche) and lysis kinetics were determined by absorbance at λ_595_nm in the presence of 0, 0.1, 0.25, 0.5, 1, 2, 3, 4 and 5 μg/ml of HL (L8402 –Sigma Aldrich) at 25°C for 2 h. rNm-ACPI, rNm-ACPII and rNg-ACP protein inhibition activity of HL was determined by dose response (0–0.5 μg protein/ml) experiments. Lysis of a bacterial suspension of *M*. *lysodeikticus* cells (1 mg/ml) was monitored over time (every 5 min for 2 h and a final end point of 24 h incubation) by measuring the change in optical density at λ_595_nm in the presence or absence of 2 μg/ml of HL in PPB, at 25°C. Specific activity of rNm-ACPI and rNm-ACPII was demonstrated by adding to the reaction a 1/16 dilution of pooled, decomplemented murine antisera raised against the recombinant protein delivered in the different formulations, as well as the corresponding sham sera and NMS as negative controls. The same procedure was repeated with rNm-MIP, which served as a negative control.

For testing if HL antimicrobial activity was responsible for *M*.*lysodiekticus* cell death, HL was boiled for 1 h and lysis kinetic experiments were done using the same conditions described above, comparing boiled HL with untreated HL and control (no HL).

### Sensitivity of live *Neisseria* spp. to lysozyme

Wild-type *N*.*meningitidis* MC58, MC161, *N*. *gonorrhoeae* FA1090 and *N*. *lactamica*, and *acp* knock-out and complemented strains, were grown overnight at 37°C with an atmosphere of 5% (v/v) CO_2_ [41] on supplemented GC agar plates with the addition of the corresponding antibiotic when necessary. Bacteria were suspended in 1 ml of supplemented GC broth, diluted 1/100 in fresh supplemented GC broth and incubated for a further 2–3 h. To the cultures was then added 250 μM IPTG, to allow expression of rNm-ACPI, rNm-ACPII or rNg-ACP proteins from a *lac* promoter on the chromosomally complemented strains. IPTG was also added to the corresponding wild type and knock-out strains, as well as to wild type *N*. *lactamica*, to ensure identical culture conditions. At an optical density (ODλ_600_nm) of ~ 0.6, cells were diluted serially in supplemented GC broth to 1 x 10^4^ CFU/ml, with and without human lactoferrin (L1294 –Sigma-Aldrich) at a final concentration of 3 mg/ml and/or 10 μg/ml of HL. It has been reported that lactoferrin is needed to permeabilize the OM of Gram-negative bacteria [64, 65], but in our study the observed effects of lysozyme on the *Neisseria* OM were similar in the presence or absence of lactoferrin [25]. For wild type *N*. *lactamica*, an assay was developed in which bacteria were incubated with and without HL (10 μg/ml) in the presence or absence of purified anti-rNm-ACPI rabbit IgG antibodies (90 μg/ml).

For both assays, culture medium samples were diluted serially in supplemented GC broth at time (t) = 0 h and at t = 8 h, plated onto GC agar plates and incubated at 37°C with an atmosphere of 5% (v/v) CO_2_ overnight. Fold changes in bacterial growth at 8 h were calculated using the following equation: N_0_/N, where N_0_ is the number of CFUs after 8 h incubation without HL, and N is the number of CFUs in the presence of HL at the same time point.

### Site-directed mutagenesis

PCR-based site directed mutagenesis was used to generate single, double and triple Nm-ACPI mutants in pET22b expression vector using the following primers:

N79Afwd 5'-CGGTGAATCTGGATAAAAGCGATGCTGTGGAAACCTTCTATGG-3';

N79Arev 5'-CCATAGAAGGTTTCCACAGCATCGCTTTTATCCAGATTCACCG-3';

Y84Afwd 5’-GCGATAATGTGGAAACCTTCGCTGGTAAAGAAGGTGGTTACG-3’;

Y84Arev 5’-CGTAACCACCTTCTTTACCAGCGAAGGTTTCCACATTATCGC-3’;

G95Afwd 5'-CTTTTACCATCCATAACAGCGGTGCCTAAAACGTAAC-3' and

G95Arev 5'-GTTACGTTTTAGGCACCGCTGTTATGGATGGTAAAAG-3'.

For each mutant, two synthetic mutagenic oligonucleotide primers (synthesized by Eurofins) were used to substitute the respective amino acid residue with alanine. The forward and reverse mutagenic primers were 37 to 43 bp long and designed to have the substitution at the centre of the sequence. PCR amplification was carried out in a PCR-thermal cycler (Prime), and subjected to the following thermal cycle reaction: 95 ^0^C for 2 min (1 cycle); 95 ^0^C for 50 s, 62 ^0^C for 50 s, 72 ^0^C for 6 min (20 cycles); 72 ^0^C for 7 min (1 cycle). Plasmids generated were incubated with DpnI enzyme (New England Biolabs) at 37 ^0^C for 2 hr and transformed into XL-10 gold ultra-competent *E*. *coli* cells for colony growth on LB-agar plates supplemented with 100 mg/ml ampicillin. The sequence of each mutant generated was confirmed by nucleotide sequencing (GATC Biotech) and subsequently expressed and purified as described for wild-type Nm-ACPI.

### Antibiotic susceptibility assay

Wild-type *Neisseria meningitidis* strains MC58 and MC161 and wild-type *Neisseria gonorrhoeae* strain FA1090 and the corresponding *Δacp* and complemented strains were grown overnight on supplemented GC agar medium and colonies suspended in pre-warmed supplemented GC broth at an ODλ_600_nm of 0.1 (equivalent to ~0.5×10^8^ CFU/mL). A 100μL aliquot of each bacterial suspension was diluted with an equal volume of medium and spread evenly onto the surface of fresh GC agar plates and allowed to dry. Etest strips with vancomycin and streptomycin (Biomerieux) were placed aseptically onto each agar plates and the plates incubated at 37°C with 5% (v/v) CO_2_. The minimum inhibitory concentration (MIC) of each antibiotic for each organism was determined following the Etest reading guide from the manufacturer.

### Measurement of membrane permeability using dye viability assay

Wild-type *Neisseria meningitidis* strains MC58 and MC161 and wild-type *Neisseria gonorrhoeae* strain FA1090 and the corresponding *Δacp* and complemented strains were grown overnight on supplemented GC agar medium and colonies suspended in 0.5mL volumes of pre-warmed supplemented GC broth. From these initial suspensions, 100μL aliquots were used to inoculate 5mL of GC broth and the cultures incubated at 37°C with shaking (200 rpm) until mid-logarithmic growth phase was reached (ODλ_600_nm >0.4). Bacteria were centrifuged (5500 g, 3 min), the pellet washed once in sterile saline (0.9% w/v NaCl) and each pellet suspended in a final volume of 1mL of saline, to which was added 3μL of a 1:1 mixture of the propidium iodide (PI, red) and Syto9 (green) dyes from the LIVE/DEAD *Bac*light Bacterial Viability Kit (Molecular Probes). Mixtures were kept at room temperature for 15 min in the dark and then 5μL volumes applied in duplicate to microscope slides under cover slips and viewed with a fluorescence microscope (Leica DMRB, Leitz). Approximately 5–6 independent fields of view were examined per sample, counting ~100 colonies per field. A minimum of n = 3 biological replicates were done per bacterium. The percentage of bacteria positive for red PI staining, indicating bacteria with permeable membranes, was calculated by dividing PI-positive bacteria by total (red and Syto9 green) bacteria.

### Statistics

Data were compared using either a two-sample t-Test or a paired t-Test, with P values <0.05 considered significant.

## Supporting information

S1 Fig Size exclusion chromatography (SEC) elution profile and SDS PAGE gels of purified *Neisseria* ACP proteins. Nm-ACP types I and II, and Ng-ACP proteins were expressed as recombinant mature soluble proteins with a C-terminal hexa-histidine tag. A) SEC profile of rNm-ACPI run at 1 ml/min in 25 mM HEPES/NaOH buffer, pH 7.5, 150 mM NaCl, on a Superdex75 HiLoad 16/600 column. An acrylamide SDS-PAGE gel of purified B) rNm-ACPI, C) rNm-ACPII and D) rNg-ACP. All three recombinant proteins are shown as a single band of *Mr* ~12.3 kDa.
(PPTX)

S2 Fig Dose response of HL enzymatic activity. Suspensions of *M*. *lysodeikticus* cells with an initial ODλ_595_nm of 1–1.2 (1 mg/ml) were treated with increasing concentrations of HL (0, 0.1, 0.25, 0.5, 1, 2, 3, 4 and 5 μg/ml) and absorbance was measured every 5 min for a period of 2 h. The symbols represent the mean values and the error bars the standard error of the means from three independent experiments. Data were compared using a paired t-Test and the asterisk (*) denotes the concentration of HL chosen for the kinetic assays.
(PPTX)

S3 Fig Analyses of the quality and specificity of murine anti-Nm-ACP sera. **A) Fluorescence-Activated Cell Sorting (FACS) analysis.** Murine antisera raised against rNm-ACPI and rNm-ACPII delivered in saline solution, Al(OH)_3_ or liposomes formulations were reacted against Nm-ACPI or Nm-ACPII expressed on the surface of MC58 or MC161 wild-type meningococci strains respectively, as demonstrated by FACS analysis. The area within the black lines show no reactivity of wild-type MC58 or MC161 bacteria with murine sham-immunised serum (1/10) and the area within the grey lines shows the significant FACS reactivity of murine antisera (1/10) raised against rNm-ACPI and rNm-ACPII in the various formulations. The same murine sham-immunised sera and antisera were non-reactive against the corresponding *nm-acp* isogenic knock-out strains. The numbers within each panel refer to the FITC-mean value. The asterisks (*) denotes the significant (P<0.05) and right-shifted increases in FITC-fluorescence recorded events, using a two sample *t*-Test to compare mean fluorescence values of test murine antisera against sham-immunised murine sera. Data are representative of n = 2 experiments. **B) Enzyme-Linked ImmunoSorbent Assay (ELISA).** ELISA reactivity of antisera from individual animals immunised with rNm-ACPI or rNm-ACPII in various adjuvant and delivery formulations were reacted against the pure recombinant proteins and MC58 or MC161 outer membranes (OM) preparations. The columns represent the geometric mean reciprocal ELISA titres (n = 5 animals per group) and the error bars represent the 95% confidence limits. No significant reactivity with pure recombinant proteins or homologous OM was observed with sera from sham-immunised animals or with normal mouse serum (NMS) (absorbance values OD_450_ <0.1 for serum dilutions of 1/10). **C) Western immunoblotting.** Pooled antisera (1/100 dilution) were reacted against wild-type MC58-OM and MC161-OM preparations in western blot. Nm-ACPI and Nm-ACPII were recognised as a single band of Mr ~12.5 kDa (identified by the arrow). All sham immunisation sera and NMS were non-reactive. All three assays (**A**, **B** and **C**) not only demonstrated specific humoral immune response of murine antisera, but also showed strong positive cross-reactivity with either antigen types.
(PPTX)

S4 Fig Alignment of the NEIS2075 (NMB2095) amino acid sequences encoded by *N*.*meningitidis* Allele I (Nm-ACPI), Allele II (Nm-ACPII) and *N*.*gonorrhoeae* Allele 10 (Ng-ACP) to compare the Loop 4 binding interface. Amino acid sequence alignments were generated using Clustal Omega (http://www.ebi.ac.uk/Tools/msa/clustalo/). The position of the Loop 4 putative binding interface for ACP interactions with lysozyme is shown in the box and amino acid differences are highlighted in red. * (asterisk) denotes fully conserved amino acid residue;: (colon) indicates conservation between groups of strongly similar properties;. (period) denotes conservation between groups of weakly similar properties.
(DOCX)

S5 Fig Human neutrophil lysozyme inhibitory activity of rNm-ACPII. The same assay parameters used with rNm-ACPI protein (Fig 4 and Fig 5) were also used to examine the dose-dependent kinetics of rNm-ACPII inhibition of HL-induced lysis of *M*.*lysodeikticus*. **A)** Lysis of a 1 mg/ml *Micrococcus lysodeikticus* cell suspension in the absence or in the presence of increasing concentrations of rNm-ACPII and 2 μg/ml of HL. The curves represent the mean absorbance (ODλ_595_nm) and the error bars represent the corresponding standard error of the mean (SEM) of three independent experiments. Data were compared with a paired t-Test and the asterisks (*) denote significant difference (P<0.05) in ODλ_595_nm in comparison to the control treatment with HL only without rNm-ACPII (0 μg/ml). **B)** Estimated percentage lysis for each test condition after 2 h and 24 h incubation. The columns represent the mean (from n = 3 independent experiments) and the error bars represent the corresponding SEM. Data were compared with a two-sample t-Test and the asterisks (*) denote significant difference (P<0.05) compared to the control without rNm-ACPII.
(PPTX)

S6 Fig Antibodies to rNm-ACPII prevent rNm-ACPII from inhibiting HL lytic activity on *M*. *lysodeikticus* cells. HL inhibitory activity by rNm-ACPII (0.5 μg/ml) was analysed as a reduction in ODλ_595_nm (Absorbance, Abs) against time of a *M*. *lysodeikticus* cell suspension (1 mg/ml) in the presence or absence of **A)** decomplemented murine antisera raised against rNm-ACPI protein in different formulations and **B)** sera from sham immunised mice. Normal mouse serum (NMS) and addition of a recombinant heterologous rNm-MIP protein were included as negative control. The symbols represent the mean absorbance (ODλ_595_nm) (from n = 3 independent experiments) and the error bars represent the corresponding standard error of the mean (SEM). Data were compared with a paired t-Test and the asterisks (*) denote significant inhibition (P<0.05) of rNm-ACPII function by anti-rNm-ACPII sera, compared to treatment without antisera. **C)** Determination of percentage of *M*. *lysodeikticus* cell lysis for each test condition is shown in **A)** and **B)** after 2 h incubation. The columns represent the mean % lysis (from n = 3 independent experiments) and the error bars represent the corresponding SEM. Ab(Lip-II), Ab(Al-II) and Ab(Sal-II) refer to decomplemented, pooled (n = 5) murine sera raised against rNm-ACPII delivered in liposomes, Al(OH)_3_ or saline solution, respectively. Ab_cont.Lip, Ab_cont.Al and Ab_cont.Sal refer to the corresponding sham immunised decomplemented, pooled (n = 5) murine sera.
(PPTX)

S7 Fig Selected region of 800MHz ^1^H-^15^N HSQC spectra of ^15^N-Nm-ACPI, alone and in complex with Hewl. The concentration of ^15^N-Nm-ACPI was 0.1 mM and Hewl was added to a final concentration of 0.01mM. Spectra were collected in sodium phosphate pH 6.5, 25°C. The spectrum of ^15^N-Nm-ACPI alone is in black, and the ^15^N-Nm-ACPI: Hewl complex is in red. Selected peaks which change on binding of Hewl are labelled.
(PPTX)

S8 Fig Alignment of non-redundant NEIS2075 (NMB2095) amino acid sequences for all *Neisseria* spp. isolates in the PubMLST database (http://pubmlst org/perl/bigsdb/bigsdb pl?db=pubmlst_neisseria_isolates). Database was accessed 19-12-2016. Amino acid sequence alignments were generated using Clustal Omega (http://www.ebi.ac.uk/Tools/msa/clustalo/) and a dendrogram was then assembled using the non-redundant sequences with Jalview 2.9 (www.jalview.org). A denotes Allele. Asterisk (*) denotes fully conserved amino acid residue;: (colon) indicates conservation between groups of strongly similar properties;. (period) denotes conservation between groups of weakly similar properties.
(DOCX)

S1 Table Analysis of NEIS2075 (NMB2095) alleles and number of isolates per *Neisseria* spp. Data are collated from (http://pubmlst.org/perl/bigsdb/bigsdb.pl?db=pubmlst_neisseria_isolates). Database accessed on 19-12-2016 displayed 153 allelic loci generating 43 non-redundant proteins, within a total population of 12483 identified isolates. Numbers in parentheses indicate that the alleles produce proteins with identical amino acid sequences. Alleles 1, 2, 6 and 10 are the most represented ones (highlighted in grey), with Allele 2 recording the highest number of isolates. Alleles 1 and 2 are the ones represented by the highest number of different pathogenic and commensal species (highlighted in grey). No identified species was reported for alleles 25, 40–43, 66–68, 71, 72, 76–78, 96, 97, 99, 100, 104–106, 111, 112, 114–145 and 147. No NEIS2075 allele information was available for commensal species *N*. *animalis*, *N*. *animaloris*, *N*. *canis*, *N*. *dentiae*, *N*. *elongata*, *N*. *elongata* subsp. *elongata*, *N*. *elongata* subsp. *glycolytica*, *N*. *elongata* subsp. *nitroreducens*, *N*. *flavescens*, *N*. *mucosa*, *N*. *musculi*, *N*. *perflava*, *N*. *shayeganii*, *N*. *sicca*, *N*. *subflava*, *N*. *wadsworthii*, *N*. *weaveri* and *N*. *zoodegmatis*.
(DOCX)

S2 Table Percentage difference in Hewl inhibition of mutants compared to wild-type ACP. The ability of wild-type Nm-ACPI and N79A, Y84A and G95A single, double and triple mutants to inhibit lysozyme was determined using the fluorescein labelled peptidoglycan substrate from *Micrococcus lysodeikticus* (EnzChek Lysozyme assay kit). The difference in lysozyme inhibition of each mutant was compared to wild-type Nm-ACPI at the initial rate period.
(DOCX)
