# Supplementary figures and images for: Structure of the Neisseria Adhesin Complex Protein (ACP) and its role as a novel lysozyme inhibitor
Source: PLoS Pathog. 2017 Jun 29;13(6):e1006448. doi: 10.1371/journal.ppat.1006448 (PMC5507604; doi:10.1371/journal.ppat.1006448)

## Slide 1
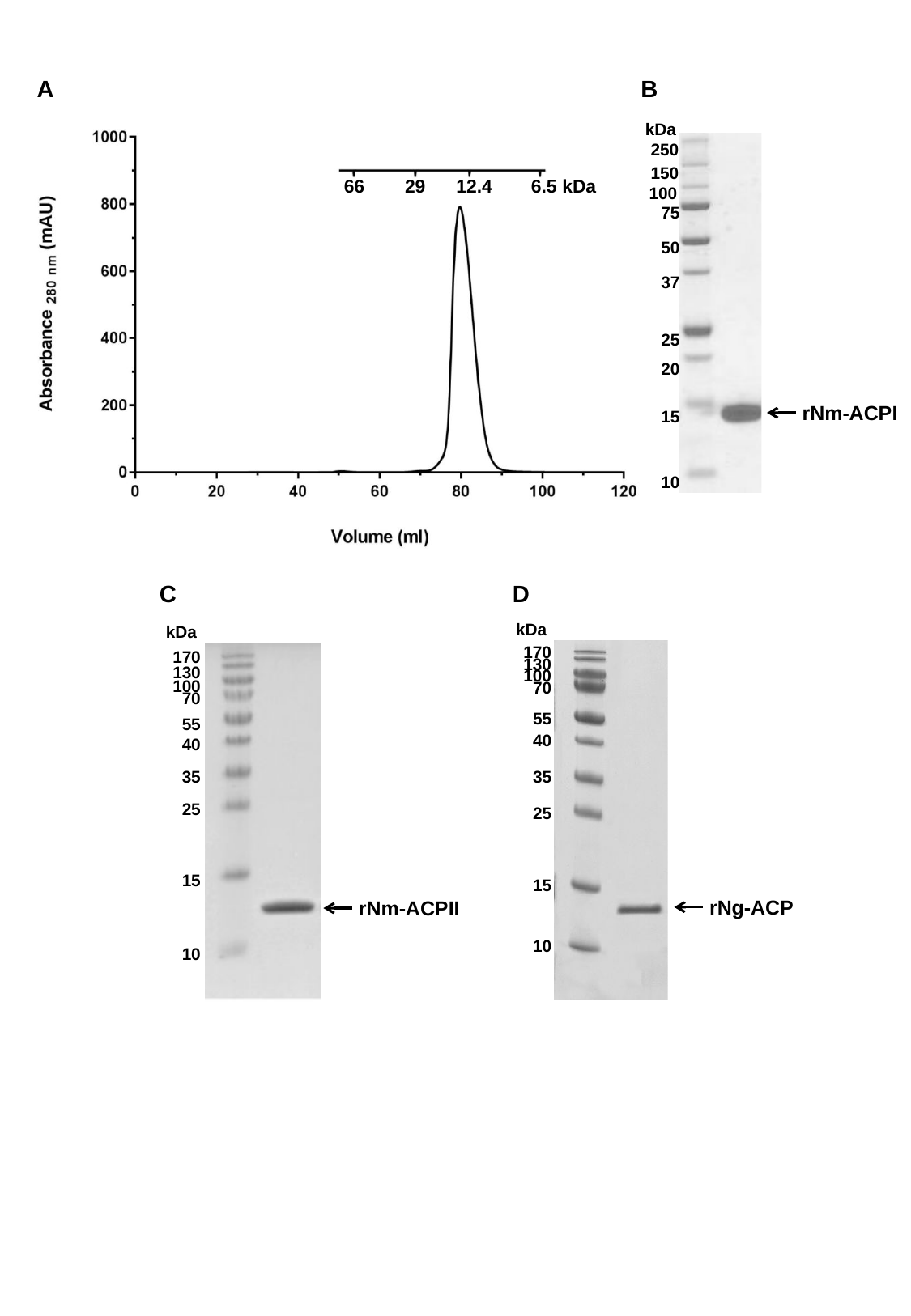

A
B
kDa
250
150
100
75
50
37
25
20
rNm-ACPI
15
10
12.4
6.5 kDa
29
66
C
kDa
170
130
100
70
55
40
35
25
15
rNm-ACPII
10
D
kDa
170
130
100
70
55
40
35
25
15
rNg-ACP
10

Supplement: S1 Fig — Nm-ACP types I and II, and Ng-ACP proteins were expressed as recombinant mature soluble proteins with a C-terminal hexa-histidine tag. A) SEC profile of rNm-ACPI run at 1 ml/min in 25 mM HEPES/NaOH buffer, pH 7.5, 150 mM NaCl, on a Superdex75 HiLoad 16/600 column. An acrylamide SDS-PAGE gel of purified B) rNm-ACPI, C) rNm-ACPII and D) rNg-ACP. All three recombinant proteins are shown as a single band of Mr ~12.3 kDa. (PPTX) [file ppat.1006448.s001.pptx]

## Slide 1
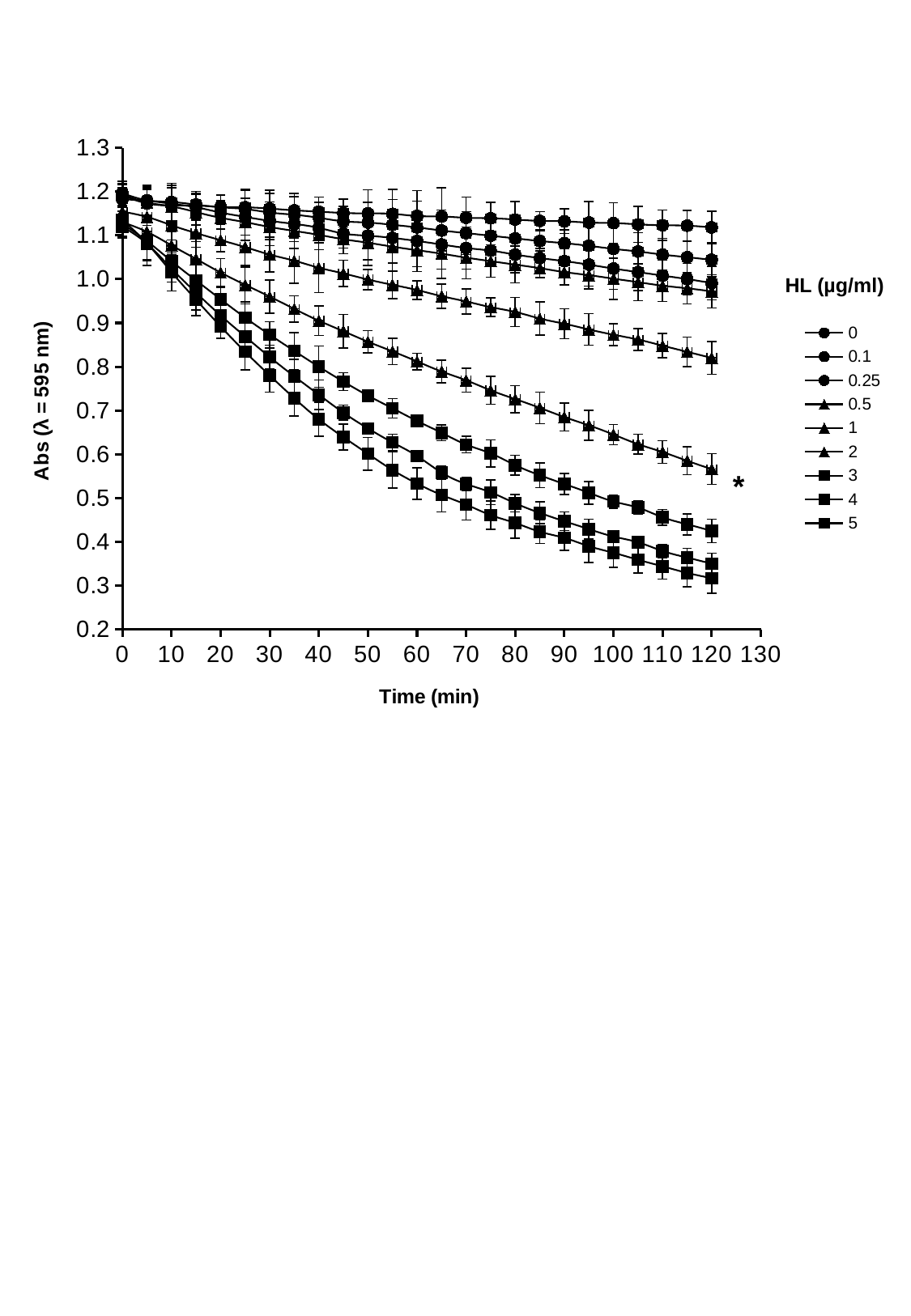

### Chart
| Category | | | | | | | | | |
|---|---|---|---|---|---|---|---|---|---|HL (µg/ml)
*

Supplement: S2 Fig — Suspensions of M. lysodeikticus cells with an initial ODλ595nm of 1–1.2 (1 mg/ml) were treated with increasing concentrations of HL (0, 0.1, 0.25, 0.5, 1, 2, 3, 4 and 5 μg/ml) and absorbance was measured every 5 min for a period of 2 h. The symbols represent the mean values and the error bars the standard error of the means from three independent experiments. Data were compared using a paired t-Test and the asterisk (*) denotes the concentration of HL chosen for the kinetic assays. (PPTX) [file ppat.1006448.s002.pptx]

## Slide 1
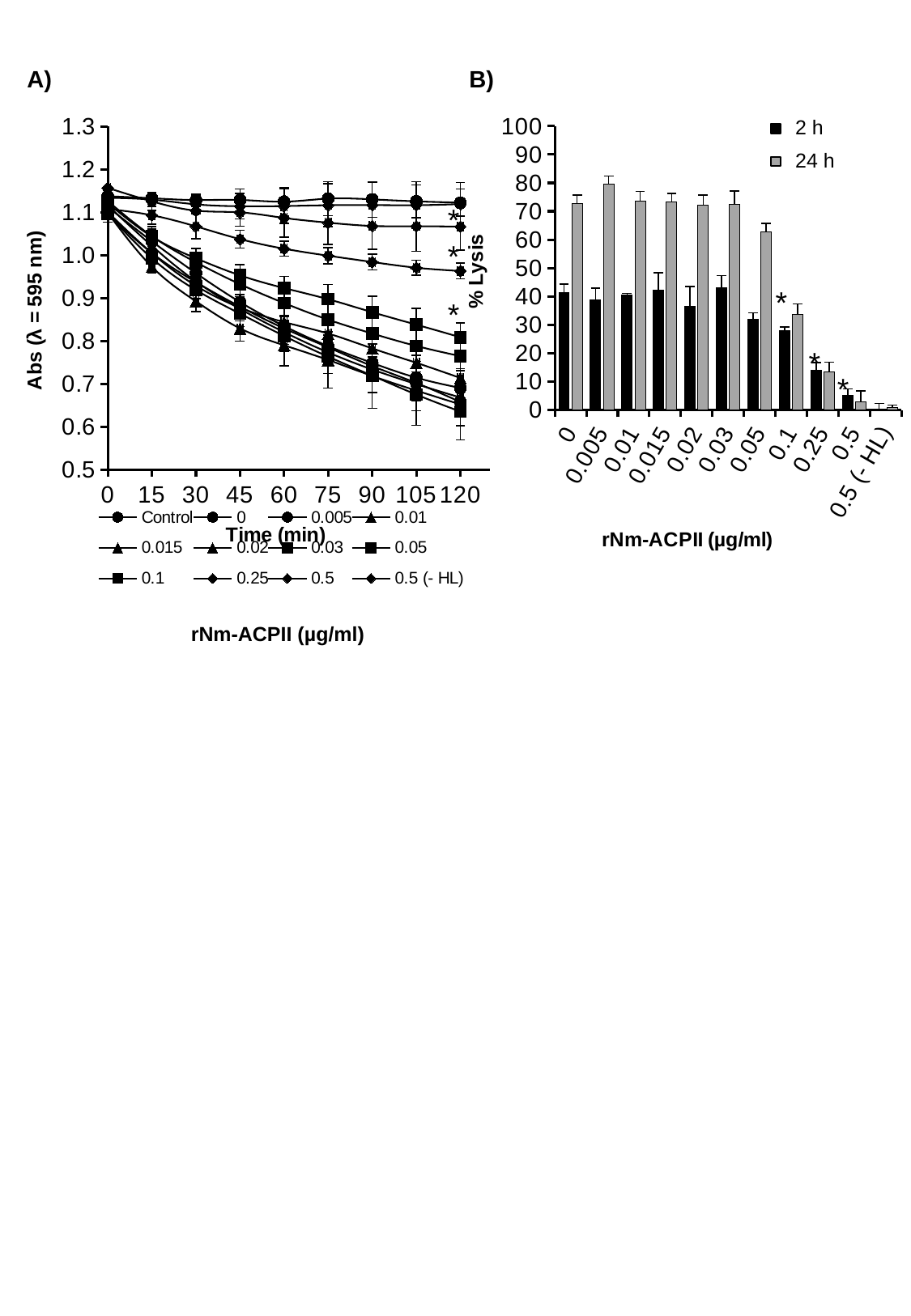

A)
### Chart
| Category | | | | | | | | | | | | |
|---|---|---|---|---|---|---|---|---|---|---|---|---|rNm-ACPII (µg/ml)
B)
### Chart
| Category | | |
|---|---|---|
| 0 | 41.27426618716691 | 72.80749030445706 |
| 0.005 | 38.66375066527201 | 79.58723919752111 |
| 0.01 | 40.43833137961064 | 73.50652536513552 |
| 0.015 | 42.06583714573431 | 73.142481263221 |
| 0.02 | 36.38731201036211 | 72.16708879854649 |
| 0.03 | 43.17853097986401 | 72.29663361363599 |
| 0.05 | 31.859376913584033 | 62.721772357633974 |
| 0.1 | 27.982906799203324 | 33.6616428208792 |
| 0.25 | 14.07788174462018 | 13.41584476523002 |
| 0.5 | 5.107992597642 | 2.9711155499110475 |
| 0.5 (- HL) | 0.38032549964665446 | 0.9335343475035008 |2 h
24 h
*
*
*
*
*
*

Supplement: S5 Fig — The same assay parameters used with rNm-ACPI protein (Fig 4 and Fig 5) were also used to examine the dose-dependent kinetics of rNm-ACPII inhibition of HL-induced lysis of M.lysodeikticus. A) Lysis of a 1 mg/ml Micrococcus lysodeikticus cell suspension in the absence or in the presence of increasing concentrations of rNm-ACPII and 2 μg/ml of HL. The curves represent the mean absorbance (ODλ595nm) and the error bars represent the corresponding standard error of the mean (SEM) of three independent experiments. Data were compared with a paired t-Test and the asterisks (*) denote significant difference (P<0.05) in ODλ595nm in comparison to the control treatment with HL only without rNm-ACPII (0 μg/ml). B) Estimated percentage lysis for each test condition after 2 h and 24 h incubation. The columns represent the mean (from n = 3 independent experiments) and the error bars represent the corresponding SEM. Data were compared with a two-sample t-Test and the asterisks (*) denote significant difference (P<0.05) compared to the control without rNm-ACPII. (PPTX) [file ppat.1006448.s005.pptx]

## Slide 1
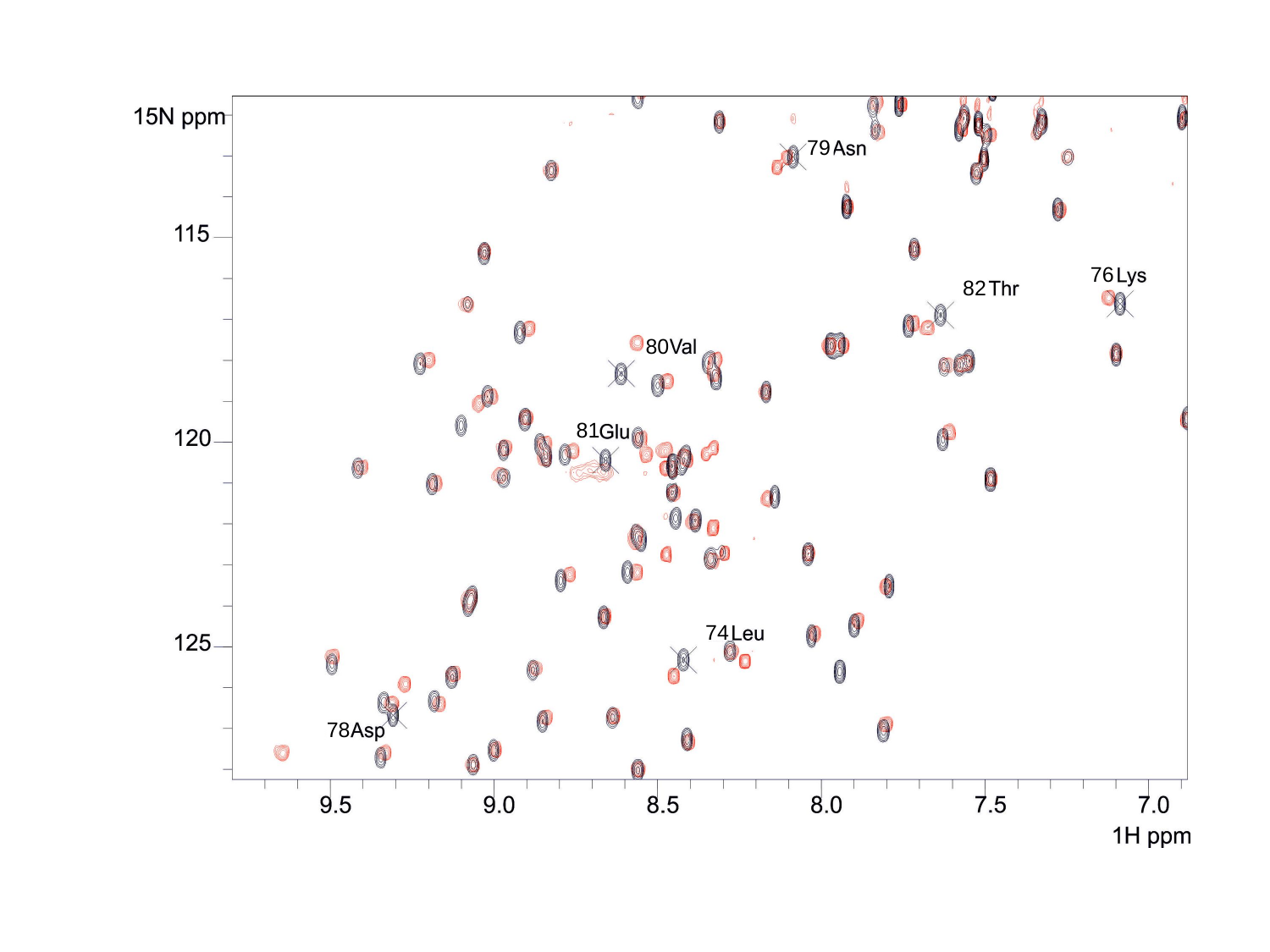

79
76
82
80
81
74
78

Supplement: S7 Fig — The concentration of 15N-Nm-ACPI was 0.1 mM and Hewl was added to a final concentration of 0.01mM. Spectra were collected in sodium phosphate pH 6.5, 25°C. The spectrum of 15N-Nm-ACPI alone is in black, and the 15N-Nm-ACPI: Hewl complex is in red. Selected peaks which change on binding of Hewl are labelled. (PPTX) [file ppat.1006448.s007.pptx]
