# Supplementary material for: Structure of the Neisseria Adhesin Complex Protein (ACP) and its role as a novel lysozyme inhibitor
Source: PLoS Pathog. 2017 Jun 29;13(6):e1006448. doi: 10.1371/journal.ppat.1006448 (PMC5507604; doi:10.1371/journal.ppat.1006448)
Supplement: S3 Fig — A) Fluorescence-Activated Cell Sorting (FACS) analysis. Murine antisera raised against rNm-ACPI and rNm-ACPII delivered in saline solution, Al(OH)3 or liposomes formulations were reacted against Nm-ACPI or Nm-ACPII expressed on the surface of MC58 or MC161 wild-type meningococci strains respectively, as demonstrated by FACS analysis. The area within the black lines show no reactivity of wild-type MC58 or MC161 bacteria with murine sham-immunised serum (1/10) and the area within the grey lines shows the significant FACS reactivity of murine antisera (1/10) raised against rNm-ACPI and rNm-ACPII in the various formulations. The same murine sham-immunised sera and antisera were non-reactive against the corresponding nm-acp isogenic knock-out strains. The numbers within each panel refer to the FITC-mean value. The asterisks (*) denotes the significant (P<0.05) and right-shifted increases in FITC-fluorescence recorded events, using a two sample t-Test to compare mean fluorescence values of test murine antisera against sham-immunised murine sera. Data are representative of n = 2 experiments. B) Enzyme-Linked ImmunoSorbent Assay (ELISA). ELISA reactivity of antisera from individual animals immunised with rNm-ACPI or rNm-ACPII in various adjuvant and delivery formulations were reacted against the pure recombinant proteins and MC58 or MC161 outer membranes (OM) preparations. The columns represent the geometric mean reciprocal ELISA titres (n = 5 animals per group) and the error bars represent the 95% confidence limits. No significant reactivity with pure recombinant proteins or homologous OM was observed with sera from sham-immunised animals or with normal mouse serum (NMS) (absorbance values OD450 <0.1 for serum dilutions of 1/10). C) Western immunoblotting. Pooled antisera (1/100 dilution) were reacted against wild-type MC58-OM and MC161-OM preparations in western blot. Nm-ACPI and Nm-ACPII were recognised as a single band of Mr ~12.5 kDa (identified by the arrow). All sham [file ppat.1006448.s003.pptx]

## Slide 1
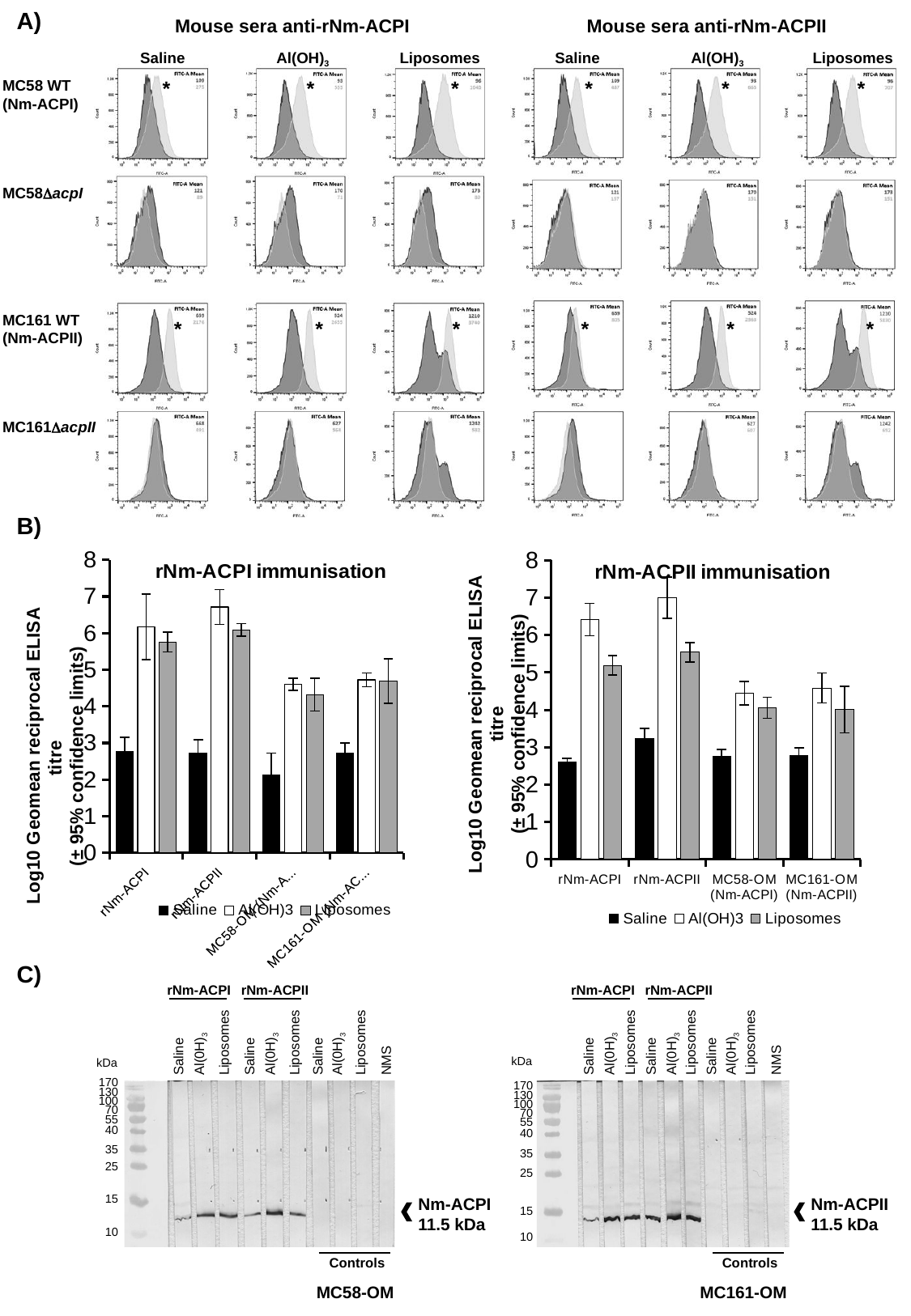

A)
Mouse sera anti-rNm-ACPI
Mouse sera anti-rNm-ACPII
Saline
Al(OH)3
Liposomes
Saline
Al(OH)3
Liposomes
MC58 WT
(Nm-ACPI)
*
*
*
*
*
*
MC58ΔacpI
MC161 WT
(Nm-ACPII)
*
*
*
*
*
*
MC161ΔacpII
B)
### Chart: rNm-ACPI immunisation
| Category | Saline | Al(OH)3 | Liposomes |
|---|---|---|---|
| rNm-ACPI | 2.758886477004609 | 6.170038629063807 | 5.756277313531507 |
| rNm-ACPII | 2.7321380935087425 | 6.715873435945856 | 6.08322244608461 |
| MC58-OM (Nm-ACPI) | 2.1168006444071135 | 4.597991044248267 | 4.317746726343601 |
| MC161-OM (Nm-ACPII) | 2.7172348557499437 | 4.7233824574370855 | 4.6912632002595345 |
### Chart: rNm-ACPII immunisation
| Category | Saline | Al(OH)3 | Liposomes |
|---|---|---|---|
| rNm-ACPI | 2.6011442191028307 | 6.415563436258631 | 5.190861672194906 |
| rNm-ACPII | 3.2338930379993807 | 7.000415821511727 | 5.538000799564066 |
| MC58-OM (Nm-ACPI) | 2.763220189460349 | 4.446048071306938 | 4.054900792535847 |
| MC161-OM (Nm-ACPII) | 2.789105537407829 | 4.581081141830488 | 4.007320875101587 |C)
rNm-ACPI
rNm-ACPII
Liposomes
Liposomes
Liposomes
Al(0H)3
Al(0H)3
Al(0H)3
Saline
Saline
Saline
NMS
kDa
170
130
100
70
55
40
35
25
Nm-ACPII
11.5 kDa
15
10
Controls
MC161-OM
rNm-ACPI
rNm-ACPII
Liposomes
Liposomes
Liposomes
Al(0H)3
Al(0H)3
Al(0H)3
Saline
Saline
Saline
NMS
kDa
170
130
100
70
55
40
35
25
15
Nm-ACPI
11.5 kDa
10
Controls
MC58-OM
