# Supplementary material for: Structure of the Neisseria Adhesin Complex Protein (ACP) and its role as a novel lysozyme inhibitor
Source: PLoS Pathog. 2017 Jun 29;13(6):e1006448. doi: 10.1371/journal.ppat.1006448 (PMC5507604; doi:10.1371/journal.ppat.1006448)
Supplement: S4 Fig — Amino acid sequence alignments were generated using Clustal Omega (http://www.ebi.ac.uk/Tools/msa/clustalo/). The position of the Loop 4 putative binding interface for ACP interactions with lysozyme is shown in the box and amino acid differences are highlighted in red. * (asterisk) denotes fully conserved amino acid residue;: (colon) indicates conservation between groups of strongly similar properties;. (period) denotes conservation between groups of weakly similar properties. (DOCX) [file ppat.1006448.s004.docx]

Allele 1 (MC58) MKLLTTAILSSAIALSSMAAAAGTDNPTVAKKTVSYVCQQGKKVKVTYGFNKQGLTTYAS 60

Allele 2 (MC161) MKLLTTAILSSAIALSSMAAAAGTNNPTVAKKTVSYVCQQGKKVKVTYGFNKQGLTTYAS 60

Allele 10(FA1090) MKLLTTAILSSAIALSSMA-AAGTDNPTVAKKTVSYVCQQGKKVKVTYGFNKQGLTTYAS 59

******************* ****:***********************************

***Loop 4***

Allele 1 (MC58) AVINGKRVQMPVNLDKSDNVETFYGKEGGYVLGTGVMDGKSYRKQPIMITAPDNQIVFKDCSPR 124

Allele 2 (MC161) AVINGKRVQMPVNLDKSDNVETFYGKEGGYVLGTGVMDGKSYRKQPIMITAPDNQIVFKDCSPR 124

Allele 10(FA1090) AVINGKRVQMPINLDKSDNMDTFYGKEGGYVLSTGAMDSKSYRKQPIMITAPDNQIVFKDCSPR 123

***********:*******::***********.**.**.*************************
