# Supplementary material for: Structure of the Neisseria Adhesin Complex Protein (ACP) and its role as a novel lysozyme inhibitor
Source: PLoS Pathog. 2017 Jun 29;13(6):e1006448. doi: 10.1371/journal.ppat.1006448 (PMC5507604; doi:10.1371/journal.ppat.1006448)
Supplement: S6 Fig — HL inhibitory activity by rNm-ACPII (0.5 μg/ml) was analysed as a reduction in ODλ595nm (Absorbance, Abs) against time of a M. lysodeikticus cell suspension (1 mg/ml) in the presence or absence of A) decomplemented murine antisera raised against rNm-ACPI protein in different formulations and B) sera from sham immunised mice. Normal mouse serum (NMS) and addition of a recombinant heterologous rNm-MIP protein were included as negative control. The symbols represent the mean absorbance (ODλ595nm) (from n = 3 independent experiments) and the error bars represent the corresponding standard error of the mean (SEM). Data were compared with a paired t-Test and the asterisks (*) denote significant inhibition (P<0.05) of rNm-ACPII function by anti-rNm-ACPII sera, compared to treatment without antisera. C) Determination of percentage of M. lysodeikticus cell lysis for each test condition is shown in A) and B) after 2 h incubation. The columns represent the mean % lysis (from n = 3 independent experiments) and the error bars represent the corresponding SEM. Ab(Lip-II), Ab(Al-II) and Ab(Sal-II) refer to decomplemented, pooled (n = 5) murine sera raised against rNm-ACPII delivered in liposomes, Al(OH)3 or saline solution, respectively. Ab_cont.Lip, Ab_cont.Al and Ab_cont.Sal refer to the corresponding sham immunised decomplemented, pooled (n = 5) murine sera. (PPTX) [file ppat.1006448.s006.pptx]

## Slide 1
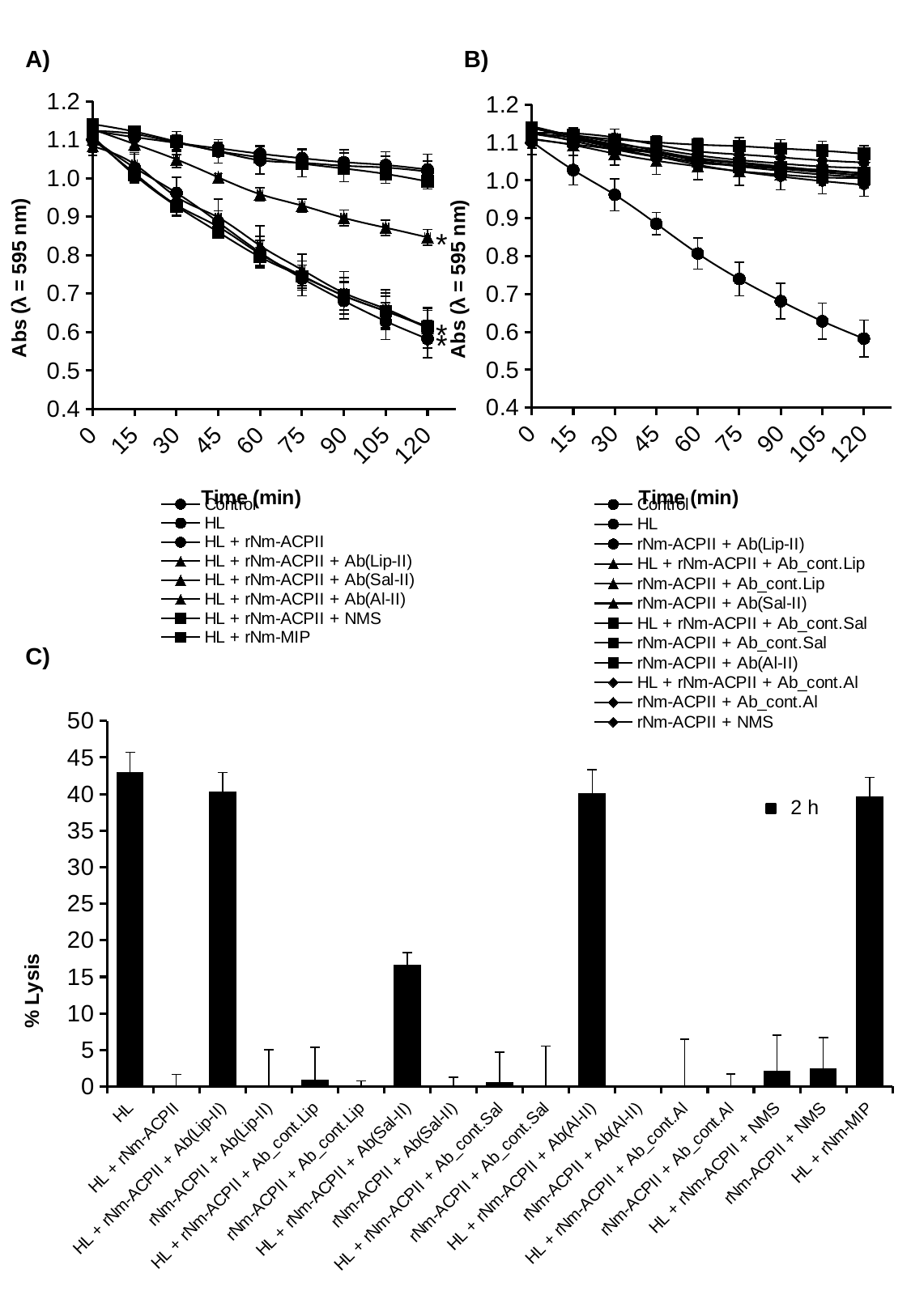

A)
B)
### Chart
| Category | Control | HL | HL + rNm-ACPII | HL + rNm-ACPII + Ab(Lip-II) | HL + rNm-ACPII + Ab(Sal-II) | HL + rNm-ACPII + Ab(Al-II) | HL + rNm-ACPII + NMS | HL + rNm-MIP |
|---|---|---|---|---|---|---|---|---|
### Chart
| Category | Control | HL | rNm-ACPII + Ab(Lip-II) | HL + rNm-ACPII + Ab_cont.Lip | rNm-ACPII + Ab_cont.Lip | rNm-ACPII + Ab(Sal-II) | HL + rNm-ACPII + Ab_cont.Sal | rNm-ACPII + Ab_cont.Sal | rNm-ACPII + Ab(Al-II) | HL + rNm-ACPII + Ab_cont.Al | rNm-ACPII + Ab_cont.Al | rNm-ACPII + NMS |
|---|---|---|---|---|---|---|---|---|---|---|---|---|*
*
*
C)
### Chart
| Category | |
|---|---|
| HL | 42.93323591162906 |
| HL + rNm-ACPII | -0.7313081084410271 |
| HL + rNm-ACPII + Ab(Lip-II) | 40.24743390523279 |
| rNm-ACPII + Ab(Lip-II) | -0.010392307717372281 |
| HL + rNm-ACPII + Ab_cont.Lip | 0.9009978683006684 |
| rNm-ACPII + Ab_cont.Lip | -0.18294197671797482 |
| HL + rNm-ACPII + Ab(Sal-II) | 16.646214801976303 |
| rNm-ACPII + Ab(Sal-II) | -1.7797043557225294 |
| HL + rNm-ACPII + Ab_cont.Sal | 0.49266864197539445 |
| rNm-ACPII + Ab_cont.Sal | -0.856713155360481 |
| HL + rNm-ACPII + Ab(Al-II) | 40.00737476216159 |
| rNm-ACPII + Ab(Al-II) | -5.514999838937347 |
| HL + rNm-ACPII + Ab_cont.Al | -0.6414876738037188 |
| rNm-ACPII + Ab_cont.Al | -3.3179287921414726 |
| HL + rNm-ACPII + NMS | 2.1025323404008893 |
| rNm-ACPII + NMS | 2.4806697638165693 |
| HL + rNm-MIP | 39.641047588492256 |2 h
