# Supplementary material for: Structure of the Neisseria Adhesin Complex Protein (ACP) and its role as a novel lysozyme inhibitor
Source: PLoS Pathog. 2017 Jun 29;13(6):e1006448. doi: 10.1371/journal.ppat.1006448 (PMC5507604; doi:10.1371/journal.ppat.1006448)
Supplement: S8 Fig — Database was accessed 19-12-2016. Amino acid sequence alignments were generated using Clustal Omega (http://www.ebi.ac.uk/Tools/msa/clustalo/) and a dendrogram was then assembled using the non-redundant sequences with Jalview 2.9 (www.jalview.org). A denotes Allele. Asterisk (*) denotes fully conserved amino acid residue;: (colon) indicates conservation between groups of strongly similar properties;. (period) denotes conservation between groups of weakly similar properties. (DOCX) [file ppat.1006448.s008.docx]

A48 MKLLSAVVLSSLVAFSGTAL----AETGNPTVSKKSVSYRCQQGKRINVTYGFNKQGLPN 56

A35 MKLLSAVVLSSLVAFSGTAL----AETGNPTVSKKSVSYRCQQGKRINVTYGFNKQGLPN 56

A47 MKLLSAVVLSSLVALSGTAL----AETGNPTVSKKSVSYRCQQGKRINVTYGFNKQGLPN 56

A23 ---LSAIVLASAVAVSGAA-IAKPAKISNPTVAKKSVTYRCQQGKHVTVTYGFNKQGLTT 56

A20 MKLLTTVILSSALALSGMAAA---AGISNPTVAKKTVSYVCQQGKKVKVTYGFNKQGLTT 57

A12 MKLLTTVILSSALALSGMAAA---AGTSNPTVAKKTVSYVCQQGKKVKVTYGFNKQGLTT 57

A101 MKLLTTVILSSALALSGMATA---AGTSNPTVAKKTVSYVCQQGKKVKVTYGFNKQGLTT 57

A80 MKLLTTAILSSAIALSSMAAA---AGTNNPTVAKKTVSYVCQQGKKVKVTYGFNKQGLTT 57

A36 MKLLTTAILSSAIALSSMAAAAGAAGTNNPTVAKKTVSYVCQQGKKVKVTYGFNKQGLTT 60

A30 MKLLTTAILSSAIALSSMAAA---AGTDNPTVAKKTVSYVCQQGKKVKVTYGFNKQGLTT 57

A149 MKLLTTAILSSAIALSSMAAA---AGTNNPTVAKKTVSYVCQQGKKVKVTYGFNKQGLTT 57

A29 MKLLTTAILSSAIALSSMAAA---AGTDNPTVAKKTVSYVCQQGKKVKVTYGFNKQGLTT 57

A110 MKLLTTAILSSAIALSSMAAA---AGTNNPTVAKKTVSYVCQQGKKVKVTYGFNKQGLTT 57

A107 MKLLTTAILSSAIALSSMAAA---AGTDNSTVAKKTVSYVCQQGKKVKVTYGFNKQGLTT 57

A109 MKLLTTAILSSAIALSSMAAA---AGTNNPTVAKKTVSYVCQQGKKVKVTYGFNKQGLTT 57

A103 MKLLTTAILSSAIALSSMAAA---AGTNNPTVAKKTVSYVCQQGKKVKVTYGFNKQGLTT 57

A69 MKLLTTAILSSAIALSSMAAT---AGTNNPTVAKKTVSYVCQQGKKVKVTYGFNKQGLTT 57

A90 MKLLTTAILSSAIALSSMAAA---AGTNNSTVAKKTVSYVCQQGKKVKVTYGFNKQGLTT 57

A89 MKLLTTAILSSAIALSSMAAA---AGTNNPTVAQKTVSYVCQQGKKVKVTYGFNKQGLTT 57

A79 MKLLTTAILSSAIALSSMAAA---AGTNNPTVAKKTVSYVCQQGKKVKVTYGFNKQGLTT 57

A75 MKLLTTAILSSAIALSSMAAA---AGTNNPTVAKKTVSYVCQQGKKVKVTYGFNKQGLTT 57

A64 MKLLTTVILSSAIALSSMAAA---AGTNNPTVAKKTVSYVCQQGKKVKVTYGFNKQGLTT 57

A62 MKLLTTAILSSAIALSSMAAA---AGTNNPSVAKKTVSYVCQQGKKVKVTYGFNKQGLTT 57

A53 MKLLTTAILSSAIALSSMAAA---AGTNNPTVAKKTVSYVCQQGKKVKVTYGFNKQGLTT 57

A51 MKLLTTAILSSAIALSSMAAA---AGTNNPTVAKKTVSYVCQQGKKVKVTYGFNKQGLTT 57

A38 MKLLTTAILSSAIALSSMAAA---AGTNNPTVAKKTVSYVCQQGKKVKVTYSFNKQGLTT 57

A24 MKLLTTAILSSAIALGSMAAA---AGTDNPTVAKKTVSYVCQQGKKVKVTYGFNKQGLTT 57

A2 MKLLTTAILSSAIALSSMAAA---AGTNNPTVAKKTVSYVCQQGKKVKVTYGFNKQGLTT 57

A1 MKLLTTAILSSAIALSSMAAA---AGTDNPTVAKKTVSYVCQQGKKVKVTYGFNKQGLTT 57

A21 MKLLTTAILSSAIALSSMATA---AGTDNPTVAKKTVSYVCQQGKKVKVTYGFNKQGLTT 57

A98 MKLLTTAILSSAIALSSMAAA---AGTDNPTVAKKTVSYVCQQGKKVKVTYGFNKQGLTT 57

A58 MKLLTTAILSSAIALSSMTAA---AGTDNPTVAKKTVSYVCQQGKKVKVTYGFNKQGLTT 57

A6 MKLLTTAILSSAIALSSMAAA---AGTNNPTVAKKTVSYVCQQGKKVKVTYGFNKQGLTT 57

A57 MKLLTTAILSSAIALSSMAAA---AGTDNPTVAKKTVSYVCQQGKKVKVTYGFNKQGLTT 57

A153 MKLLTTAILSSAIALSSMA-A---AGTDNPTVAKKTVSYVCQQGKKVKVPYGFNKQGLTT 56

A113 MKLLTTAILSSAIALSSMA-A---ADTDNPTVAKKTVSYVCQQGKKVKVTYGFNKQGLTT 56

A150 MKLLTTAILSSAIALSSMA-A---VGTDNPTVAKKTVSYVCQQGKKVKVTYGFNKQGLTT 56

A95 MKLLTTAILSSAIALSSMA-A---AGTDNPTVAKKTVSYVCQQGKKVKVTYGFNKQGLTT 56

A93 MKLLTTAILSSAIALSSMA-A---VGTDNPTVAKKTVSYVCQQGKKVKVTYGFNKQGLTT 56

A83 MKLLTTAILSSAIALSSMV-A---AGTDNPTVAKKTVSYVCQQGKKVKVTYGFNKQGLTT 56

A82 MKLLTTAILSSAIALSSMA-A---AGTDNPTIAKKTVSYVCQQGKKVKVTYGFNKQGLTT 56

A10 MKLLTTAILSSAIALSSMA-A---AGTDNPTVAKKTVSYVCQQGKKVKVTYGFNKQGLTT 56

A81 MKLLTTAILSSAIALSSMA-A---AGTDNPTVAKKTVSYVCQQGKKVKVTYGFNKQGLTT 56

*:: :*:* :*... . . .* ::::*:*:* *****::.* *.****** .

A48 YAVARIDGRNRTMDINLDRSDNVDTFFIDEGGYTLGTSAMSTKTYRKQPIMITSPKDEIL 116

A35 YAVARIDGRNRTMDINLDRSDNVDTFFIDEGGYTLGTGAMSTKNYRKQPIMITSPKDEIL 116

A47 YAVARIDGRNRTMDINLDRSDNVDTFFIDEGGYTLGTGAMSTKNYRKQPIMITSPKDEIL 116

A23 SASAVVDGKRRFMPIDLDRSDNADTYYGKEGGYVLSTAYMDKKTYRKQPIMITAPDDEIV 116

A20 YASAAIKGKQVQMPINLDKSDNMDTFYGKEGGYVLSTGAMDSKSYRKQPIMITAPDNQIV 117

A12 YASAAIKGKQVQMPINLDKSDNMDTFYGKEGGYVLSTGAMDSKSYRKQPIMITAPDNQIV 117

A101 YASAAIKGKQVQMPINLDKSDNMDTFYGKEGGYVLSTGAMDSKSYRKQPIMITAPDNQIV 117

A80 YASAVINGKRVQMPVNLDKSDNVETFYGKEGGYVLSTGAMDSKSYRKQPIMITAPDNQIV 117

A36 YASAVINGKRVQMPVNLDKSDNVETFYGKEGGYVLGTGVMDGKSYRKQPIMITAPDNQIV 120

A30 YAFAVINGKRVQMPVNLDKSDNVETFYGKEGGYVLGTGVMDGKSYRKQPIMITAPDNQIV 117

A149 YASAVINGKRVQMPVNLDKSDNVETFYGKEGGYVLGTGVIDGKSYRKQPIMITAPDNQIV 117

A29 YASAVINGKRVQMPVNLDKSDNVETFYGKEGGYVLGTGVMDSKSYRKQPIMITAPDNQIV 117

A110 YASAVINGKRVQMPVNLDKSDNVETFYSKEGGYVLGTGVMDGKSYRKQPIMITAPDNQIV 117

A107 YASAVINGKRVQMPVNLDKSDNVETFYGKEGGYVLGTGVMDGKSYRKQPIMITAPDNQIV 117

A109 YASAVINGKRVQMPVNLDKSDNVETFYGKEGGYVLGSGVMDGKSYRKQPIMITAPDNQIV 117

A103 YASAVINGKRVQMPVNLDKSDNVETFYGKEGGYVLGTGVMDGKSYRKQPIMITAPDSQIV 117

A69 YASAVINGKRVQMPVNLDKSDNVETFYGKEGGYVLGTGVMDGKSYRKQPIMITAPDNQIV 117

A90 YASAVINGKRVQMPVNLDKSDNVETFYGKEGGYVLGTGVMDGKSYRKQPIMITAPDNQIV 117

A89 YASAVINGKRVQMPVNLDKSDNVETFYGKEGGYVLGTGVMDGKSYRKQPIMITAPDNQIV 117

A79 YASAVINGKRVQMPVNLDKSDNVETFYGKEGGYVLGTGVMDGKSYRKQPIMITAPDNQIV 117

A75 YASAVINGKRVQMPVNLDKSDNVETFYGKEGGYVLGTGVMDGKSYRKQSIMITAPDNQIV 117

A64 YASAVINGKRVQMPVNLDKSDNVETFYGKEGGYVLGTGVMDGKSYRKQPIMITAPDNQIV 117

A62 YASAVINGKRVQMPVNLDKSDNVETFYGKEGGYVLGTGVMDGKSYRKQPIMITAPDNQIV 117

A53 YASAVINGKRVQMPVNLDKSDNVETFYGKEGGYVLGTGVMDGKSYRKQPIMITAPNNQIV 117

A51 YASAVINGKRVQMPINLDKSDNVETFYGKEGGYVLGTGVMDGKSYRKQPIMITAPDNQIV 117

A38 YASAVINGKRVQMPVNLDKSDNVETFYGKEGGYVLGTGVMDGKSYRKQPIMITAPDNQIV 117

A24 YASAVINGKRVQMPVNLDKSDNVETFYGKEGGYVLGTGVMDGKSYRKQPIMITAPDNQIV 117

A2 YASAVINGKRVQMPVNLDKSDNVETFYGKEGGYVLGTGVMDGKSYRKQPIMITAPDNQIV 117

A1 YASAVINGKRVQMPVNLDKSDNVETFYGKEGGYVLGTGVMDGKSYRKQPIMITAPDNQIV 117

A21 YASAVINGKRVQMPINLDKSDNMDTFYGKEGGYVLSTGAMDSKSYRKQPIMITAPDNQIV 117

A98 YASAVINGKRVQMPINLDKSDNMDTFYGKEGGYVLSTGEMDSKSYRKQPIMITAPDNQIV 117

A58 YASAVINGKRVQMPINLDKSDNMDTFYGKEGGYVLSTGAMDSKSYRKQPIMITAPDNQIV 117

A6 YASAVINGKRVQMPINLDKSDNMDTFYGKEGGYVLSTGAMDSKSYRKQPIMITAPDNQIV 117

A57 YASAVINGKRVQMPINLDKSDNMDTFYGKEGGYVLSTGAMDSKSYRKQPIMITAPDNQIV 117

A153 YASAVINGKRVQMPINLDKSDNMDTFYGKEGGYVLSTGAMDSKSYRKQPIMITAPDNQIV 116

A113 YASAVINGKRVQMPINLDKSDNMDTFYGKEGGYVLSTGAMDSKSYRKQPIMITAPDNQIV 116

A150 YASAVINGKRVQTPINLDKSDNMDTFYGKEGGYVLSTGAMDSKSYRKQPIMITAPDNQIV 116

A95 YASAVINGKRVQMPINLDKSDNMDTFYGKEGSYVLSTGAMDSKSYRKQPIMITAPDNQIV 116

A93 YASAVINGKRVQMPINLDKSDNMDTFYGKEGGYVLSTGAMDSKSYRKQPIMITAPDNQIV 116

A83 YASAVINGKRVQMPINLDKSDNMDTFYGKEGGYVLSTGAMDSKSYRKQPIMITAPDNQIV 116

A82 YASAVINGKRVQMPINLDKSDNMDTFYGKEGGYVLSTGAMDSKSYRKQPIMITAPDNQIV 116

A10 YASAVINGKRVQMPINLDKSDNMDTFYGKEGGYVLSTGAMDSKSYRKQPIMITAPDNQIV 116

A81 YASAVINGKRVQMPINLDKSDNMDTFYGKEGGYVLSTGAIDSKSYRKQPIMITAPDNQIV 116

* * :.*:. ::**:*** :*:: .**.*.*.:. :. *.**** ****:*..:*:

A48 FKSCTPR 123

A35 FKSCTPR 123

A47 FKSCTPR 123

A23 LKDCSPR 123

A20 FKDCSPR 124

A12 FKDCSPR 124

A101 FKDCSPR 124

A80 FKDCSPR 124

A36 FKDCSPR 127

A30 FKDCSPR 124

A149 FKDCSPR 124

A29 FKDCSPR 124

A110 FKDCSPR 124

A107 FKDCSPR 124

A109 FKDCSPR 124

A103 FKDCSPR 124

A69 FKDCSPR 124

A90 FKDCSPR 124

A89 FKDCSPR 124

A79 FKNCSPR 124

A75 FKDCSPR 124

A64 FKDCSPR 124

A62 FKDCSPR 124

A53 FKDCSPR 124

A51 FKDCSPR 124

A38 FKDCSPR 124

A24 FKDCSPR 124

A2 FKDCSPR 124

A1 FKDCSPR 124

A21 FKDCSPR 124

A98 FKDCSPR 124

A58 FKDCSPR 124

A6 FKDCSPR 124

A57 FKDCSPR 124

A153 FKDCSPR 123

A113 FKDCSPR 123

A150 FKDCSPR 123

A95 FKDCSPR 123

A93 FKDCSPR 123

A83 FKDCSPR 123

A82 FKDCSPR 123

A10 FKDCSPR 123

A81 FKDCSPR 123

:*.*:***

**S8 Fig. Alignment of non-redundant NEIS2075 (NMB2095) amino acid sequences for all *Neisseria* spp. isolates in the PubMLST database (**[http://pubmlst‑org/perl/bigsdb/bigsdb‑pl?db=pubmlst_neisseria_isolates](http://pubmlst.org/perl/bigsdb/bigsdb.pl?db=pubmlst_neisseria_isolates)**).** Database was accessed 19-12-2016. Amino acid sequence alignments were generated using Clustal Omega (<http://www.ebi.ac.uk/Tools/msa/clustalo/>) and a dendrogram was then assembled using the non-redundant sequences with Jalview 2.9 ([www.jalview.org](http://www.jalview.org)).

A denotes Allele. * (asterisk) denotes fully conserved amino acid residue; **:** (colon) indicates conservation between groups of strongly similar properties; **.** (period) denotes conservation between groups of weakly similar properties.
