# Supplementary material for: Structure of the Neisseria Adhesin Complex Protein (ACP) and its role as a novel lysozyme inhibitor
Source: PLoS Pathog. 2017 Jun 29;13(6):e1006448. doi: 10.1371/journal.ppat.1006448 (PMC5507604; doi:10.1371/journal.ppat.1006448)
Supplement: S1 Table — Data are collated from (http://pubmlst.org/perl/bigsdb/bigsdb.pl?db=pubmlst_neisseria_isolates). Database accessed on 19-12-2016 displayed 153 allelic loci generating 43 non-redundant proteins, within a total population of 12483 identified isolates. Numbers in parentheses indicate that the alleles produce proteins with identical amino acid sequences. Alleles 1, 2, 6 and 10 are the most represented ones (highlighted in grey), with Allele 2 recording the highest number of isolates. Alleles 1 and 2 are the ones represented by the highest number of different pathogenic and commensal species (highlighted in grey). No identified species was reported for alleles 25, 40–43, 66–68, 71, 72, 76–78, 96, 97, 99, 100, 104–106, 111, 112, 114–145 and 147. No NEIS2075 allele information was available for commensal species N. animalis, N. animaloris, N. canis, N. dentiae, N. elongata, N. elongata subsp. elongata, N. elongata subsp. glycolytica, N. elongata subsp. nitroreducens, N. flavescens, N. mucosa, N. musculi, N. perflava, N. shayeganii, N. sicca, N. subflava, N. wadsworthii, N. weaveri and N. zoodegmatis. (DOCX) [file ppat.1006448.s009.docx]

| **ALLELE NEIS2075 (NMB2095)** | **Pathogenic *Neisseria* spp.** | | **Commensal *Neisseria* spp.** | | | | | | **TOTAL** |
| --- | --- | --- | --- | --- | --- | --- | --- | --- | --- |
|  | ***N. meningitidis*** | ***N. gonorrhoeae*** | ***N. lactamica*** | ***N. polysaccharea*** | ***N. cinerea*** | ***N. bacilliformis*** | ***N. oralis*** | ***N. bergeri*** |  |
| **1** (+7+8+11+16+19+ 27+28+34+37+61+91 +102+108) | 570 |  | 100 | 11 |  |  |  | 1 | **682** |
| **2** (+3+4+5+9+13 +14 +15+17+18+32+33+39+45+52+54+55+56+63+65+70+73+74+84+85 +86+87 +88+92+148) | 7966 | 6 |  |  | 1 |  |  |  | **7973** |
| **6** (+59+60) |  | 553 |  |  |  |  |  |  | **553** |
| **10** (+146) |  | 3021 |  |  | 1 |  |  |  | **3022** |
| **12** (+22+31+44) |  |  |  |  | 4 |  |  |  | **4** |
| **20** |  |  |  |  | 4 |  |  |  | **4** |
| **21** |  |  |  |  | 1 |  |  |  | **1** |
| **23** (+49+50) |  |  |  |  |  |  | 3 |  | **3** |
| **24** (+26+46) | 1 |  | 39 |  |  |  |  |  | **40** |
| **29** |  |  |  | 4 |  |  |  |  | **4** |
| **30** |  |  |  | 1 |  |  |  |  | **1** |
| **35** |  |  |  |  |  | 2 |  |  | **2** |
| **36** | 5 |  |  |  |  |  |  |  | **5** |
| **38** | 3 |  |  |  |  |  |  |  | **3** |
| **47** |  |  |  |  |  | 1 |  |  | **1** |
| **48** |  |  |  |  |  | 1 |  |  | **1** |
| **51** | 1 |  |  |  |  |  |  |  | **1** |
| **53** | 1 |  |  |  |  |  |  |  | **1** |
| **57** (+151+152) |  | 4 |  |  | 1 |  |  |  | **5** |
| **58** |  |  |  |  | 1 |  |  |  | **1** |
| **62** | 3 |  |  |  |  |  |  |  | **3** |
| **64** | 1 |  |  |  |  |  |  |  | **1** |
| **69** | 2 |  |  |  |  |  |  |  | **2** |
| **75** | 1 |  |  |  |  |  |  |  | **1** |
| **79** | 1 |  |  |  |  |  |  |  | **1** |
| **80** |  | 1 |  |  |  |  |  |  | **1** |
| **81** |  | 3 |  |  |  |  |  |  | **3** |
| **ALLELE NEIS2075 (NMB2095)** | **Pathogenic *Neisseria* spp.** | | **Commensal *Neisseria* spp.** | | | | | | **TOTAL** |
|  | ***N. meningitidis*** | ***N. gonorrhoeae*** | ***N. lactamica*** | ***N. polysaccharea*** | ***N. cinerea*** | ***N. bacilliformis*** | ***N. oralis*** | ***N. bergeri*** |  |
| **82** |  | 5 |  |  |  |  |  |  | **5** |
| **83** |  | 1 |  |  |  |  |  |  | **1** |
| **89** | 1 |  |  |  |  |  |  |  | **1** |
| **90** | 1 |  |  |  |  |  |  |  | **1** |
| **93** (+94) |  | 123 |  |  |  |  |  |  | **123** |
| **95** |  | 2 |  |  |  |  |  |  | **2** |
| **98** |  |  |  | 1 |  |  |  |  | **1** |
| **101** |  |  |  |  | 1 |  |  |  | **1** |
| **103** | 1 |  |  |  |  |  |  |  | **1** |
| **107** | 7 |  |  |  |  |  |  |  | **7** |
| **109** | 1 |  |  |  |  |  |  |  | **1** |
| **110** | 1 |  |  |  |  |  |  |  | **1** |
| **113** |  | 11 |  |  |  |  |  |  | **11** |
| **149** | 1 |  |  |  |  |  |  |  | **1** |
| **150** |  | 6 |  |  |  |  |  |  | **6** |
| **153** |  | 1 |  |  |  |  |  |  | **1** |
| **TOTAL** | **8568** | **3737** | **139** | **17** | **14** | **4** | **3** | **1** | **12483** |

**S1 Table: Analysis of NEIS2075 (NMB2095) alleles and number of isolates per *Neisseria* spp.** Data are collated from <http://pubmlst.org/perl/bigsdb/bigsdb.pl?db=pubmlst_neisseria_isolates>. Database accessed on 19-12-2016 displayed 153 allelic loci generating 43 non-redundant proteins, within a total population of 12483 identified isolates. Numbers in parentheses indicate that the alleles produce proteins with identical amino acid sequences. Alleles 1, 2, 6 and 10 are the most represented ones (highlighted in grey), being allele 2 the one with the higher number of isolates reported. Alleles 1 and 2 are the ones represented by the higher number of different pathogenic and commensal species (highlighted in grey). No identified species was reported for alleles 25, 40-43, 66, 67-68, 71, 72, 76-78, 96, 97, 99, 100, 104-106, 111, 112, 114-145 and 147. No NEIS2075 allele information was available for commensal species *N. animalis, N. animaloris, N. canis, N. dentiae, N. elongata, N. elongata* subsp. *elongata, N. elongata* subsp. *glycolytica, N. elongata* subsp. *nitroreducens, N. flavescens, N. mucosa, N. musculi, N. perflava, N. shayeganii, N. sicca, N. subflava, N. wadsworthii, N. weaveri* and *N. zoodegmatis.*
