# Supplementary material for: Structure of the Neisseria Adhesin Complex Protein (ACP) and its role as a novel lysozyme inhibitor
Source: PLoS Pathog. 2017 Jun 29;13(6):e1006448. doi: 10.1371/journal.ppat.1006448 (PMC5507604; doi:10.1371/journal.ppat.1006448)
Supplement: S2 Table — The ability of wild-type Nm-ACPI and N79A, Y84A and G95A single, double and triple mutants to inhibit lysozyme was determined using the fluorescein labelled peptidoglycan substrate from Micrococcus lysodeikticus (EnzChek Lysozyme assay kit). The difference in lysozyme inhibition of each mutant was compared to wild-type Nm-ACPI at the initial rate period. (DOCX) [file ppat.1006448.s010.docx]

**S2 Table. Percentage lysozyme inhibition of wild-type ACP and mutants.**

| **Protein** | **% Lysozyme activity** |
| --- | --- |
| Hewl | 100.00 |
| WT_ACP | 4.04 |
| N79A_ACP | 8.28 |
| Y84A_ACP | 17.20 |
| G95A_ACP | 5.39 |
| N79A/Y84A_ACP | 27.41 |
| N79A/G95A_ACP | 7.48 |
| Y84A/G95A_ACP | 27.01 |
| N79A/Y84A/G95A_ACP | 38.91 |

Hewl activity was assayed using the EnzChek® Lysozyme assay kit, as described in Materials and Methods. Each reaction mixture contained 0.2mg rNm-ACPI (mutant or wild type) and 10U of Hewl in 100µl reaction volume. The reaction was allowed to proceed at 37^o^C and fluorescence readings taken every 10 mins. WT, wild-type.
